# Multi‐Step Assembly of an RNA‐Liposome Nanoparticle Formulation Revealed by Real‐Time, Single‐Particle Quantitative Imaging

**DOI:** 10.1002/advs.202414305

**Published:** 2025-01-31

**Authors:** Michael C. Chung, Hector R. Mendez‐Gomez, Dhruvkumar Soni, Reagan McGinley, Alen Zacharia, Jewel Ashbrook, Brian Stover, Adam J. Grippin, Elias J. Sayour, Juan Guan

**Affiliations:** ^1^ Division of Chemical Biology and Medicinal Chemistry College of Pharmacy University of Texas at Austin Austin TX 78712 USA; ^2^ Department of Physics University of Florida Gainesville FL 32611 USA; ^3^ Department of Neurosurgery Preston A. Wells, Jr. Center for Brain Tumor Therapy, McKnight Brain Institute, University of Florida Lillian S. Wells Gainesville FL 32610 USA; ^4^ Department of Microbiology and Cell Science University of Florida Gainesville FL 32603 USA; ^5^ Middlebury College Department of Physics McCardell Bicentennial Hall Middlebury VT 05753 USA; ^6^ Department of Pediatrics Division of Pediatric Hematology Oncology University of Florida Gainesville FL 32610 USA; ^7^ MD Anderson Cancer Center Division of Radiation Oncology University of Texas Houston TX 77030 USA

**Keywords:** cancer‐therapy, drug‐delivery, imaging, nanomaterials, nanoparticles, quantitative imaging, self‐assembly

## Abstract

Self‐assembly plays a critical role in nanoparticle‐based applications. However, it remains challenging to monitor the self‐assembly of multi‐component nanomaterials at a single‐particle level, in real‐time, with high throughput, and in a model‐independent manner. Here, multi‐color fluorescence microscopy is applied to track the assembly of both liposomes and mRNA simultaneously in clinical mRNA‐based cancer immunotherapy. Imaging reveals that the assembly occurs in discrete steps: initially, RNA adsorbs onto the liposomes; then, the RNA‐coated liposomes cluster into heterogeneous structures ranging from sub‐micrometer to tens of micrometers. The clustering process is consistent with a Smoluchowski model with a Brownian diffusion kernel. The transition between the two steps of assembly is determined by the orientation of RNA‐mediated interactions. Given the facile application of this approach and the ubiquity of the components studied, the imaging and analysis in this work are readily applied to monitor multi‐component assembly of diverse nanomaterials.

## Graphical abstract

This work uses multi‐color fluorescence imaging to dissect the dynamic self‐assembly of RNA‐Liposome complexes. The single‐particle, real‐time imaging data enables detailed modeling of this multi‐stage process, from RNA adsorption to higher‐order cluster assembly, across many different formulations and preparatory conditions. This approach enhances mechanistic understanding and rational design of RNA‐based lipoplexes and other biomaterial systems.

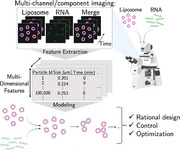

## 1 Introduction

The rational design and engineering of nanoparticles is critical for any nanoparticle‐based application; this is especially true for achieving effective nanotherapeutics and addressing challenges in modern translational medicine.^[^ ^1^, ^2^, ^3^, ^4^ ^]^ These systems are often formulated by leveraging the self‐assembly of multiple components such that bio‐compatible materials (e.g., polymers or lipid vesicles) serve as nanometer‐sized carriers to deliver therapeutic molecules (e.g., nucleic acids or small‐molecule drugs) to targeted cells or tissues.^[^ ^5^, ^6^, ^7^, ^8^, ^9^, ^10^ ^]^ This self‐assembly process ultimately determines the physicochemical properties of the resulting nanoparticles and how the formulation interacts with biological systems. Therefore, a mechanistic understanding of the self‐assembly process in a nanoparticle formulation can provide the insight necessary for control and improvement of these formulations.

However, despite the existence of a variety of methods for understanding the nano‐to‐microscale characteristics of nanoparticle formulations,^[^ ^11^ ^]^ the exact details of the self‐assembly process are often elusive due to the limitations of these methods. Specifically, these techniques do not possess all the following desirable capabilities: i) recording the dynamics of the assembly in real time, ii) simultaneously monitoring all components, iii) single‐particle resolution, iv) high throughput, v) model‐independence, and vi) minimal perturbation of the underlying system. For instance, electron microscopy techniques (e.g., Cryo‐EM), while providing impressive spatial resolution, may not monitor real‐time dynamics or faithfully preserve the morphology of nanomaterials during sample preparation, and are relatively low‐throughput. Although scattering techniques (e.g., dynamic light scattering or DLS) can monitor real‐time dynamics in nanoparticle systems, they are indirect ensemble‐averaged measurements that rely heavily on model fitting. Therefore, it is imperative to develop methods capable of monitoring the dynamic assembly of multi‐component nanoparticle systems with the aforementioned capabilities.

**Figure 1:**
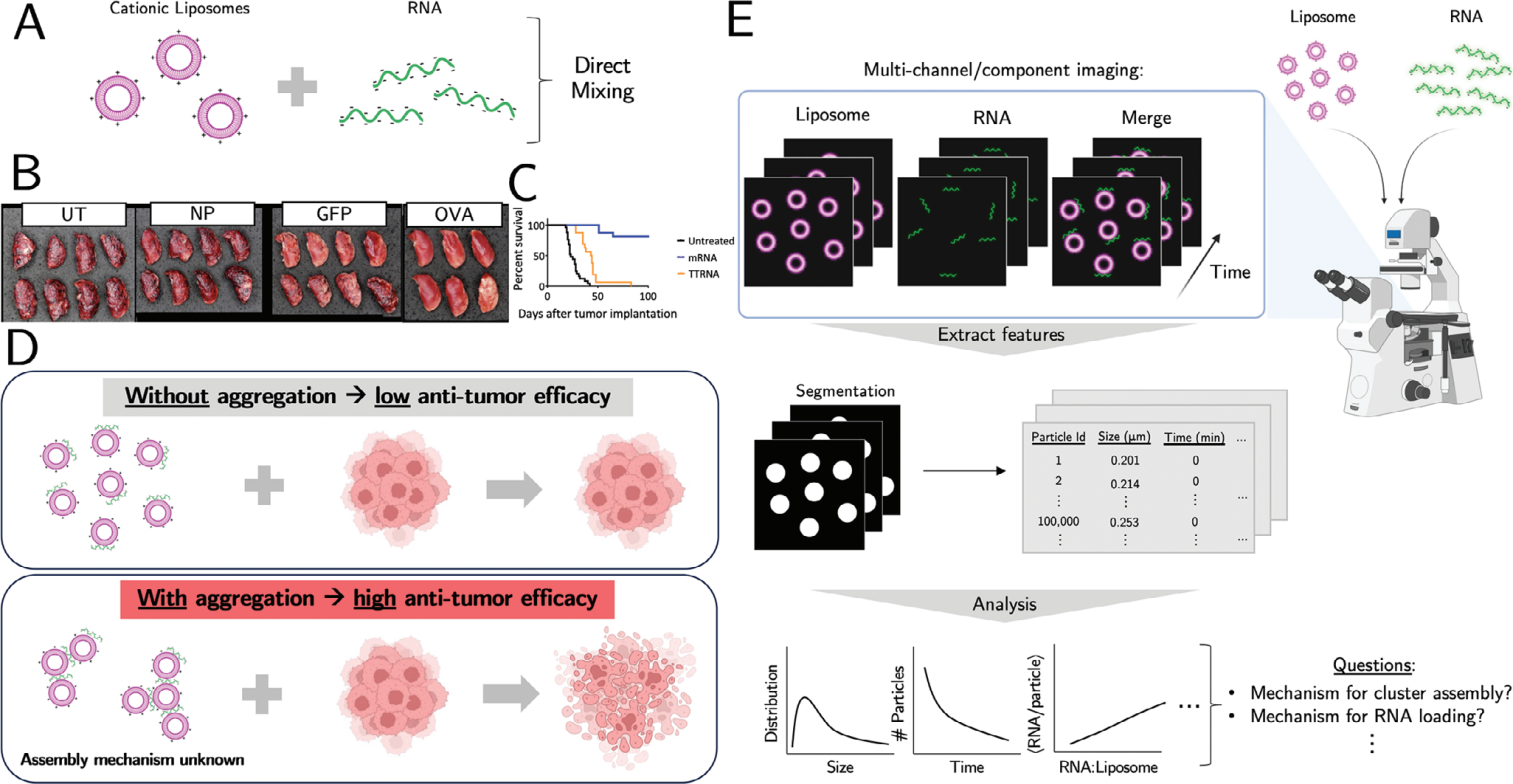
The experimental system and fluorescence imaging analysis workflow. A) To form our RNA‐liposomes, we directly mix cationic liposomes and RNA (see text for details). B) Images of pulmonary B16F10‐OVA in C57Bl/6 mice following treatment with GFP versus OVA mRNA vaccines. UT and NP denote the untreated and nanoparticles without RNA, respectively. C) Merger of 3 experiments for i.v. inoculated K7M2 in Balb/c mice (n = 8–17) i.v. treated once weekly x3 with either total tumor RNA (rRNA, tRNA, mRNA) or tumor mRNA. D) Particle aggregation in a clinical RNA‐liposome formulation (see text for details) dramatically improves anti‐tumor efficacy. However, its assembly mechanism is unknown and is the focus of this study. E) Fluorescence imaging and analysis workflow of the multi‐component RNA‐liposome system. This experimental design enables recording multi‐component, single‐particle dynamics with high throughput. A variety of dynamic features, which include size and intensity metrics, are extracted from quantitative image analysis and are used for further modeling of the assembly process.

Assessing the assembly of multi‐component nanoparticle systems is especially important in burgeoning RNA‐based cancer immunotherapy applications,^[^ ^12^, ^13^, ^14^, ^15^, ^16^, ^17^, ^18^, ^19^, ^20^ ^]^ where the physical and chemical properties of the nanomedicine composition determine the outcome of RNA delivery and ultimately the clinical efficacy.^[^ ^21^, ^22^, ^23^, ^24^, ^25^, ^26^ ^]^ In our FDA‐approved clinical trial (NCT04573140), we developed an effective nanoparticle formulation, which directly mixes cationic liposomes composed of pure 1,2‐dioleoyl‐3‐trimethylammonium‐propane (DOTAP) lipids with tumor‐derived RNA (**Figure** **1**A), for personalized cancer immunotherapy that substantially reprograms the tumor microenvironment by engaging both the innate and adaptive arms of the immune systems, culminating in therapeutic anti‐tumor potential^[^ ^27^ ^]^ (Figure 1B,C).

This is an ideal model system to study self‐assembly for several reasons. First, while the work demonstrated that nanoparticle aggregation was key to elicit the potent immune response, the detailed mechanism of the self‐assembly process leading to the aggregation is unknown (Figure 1D). Specifically, though RNA‐liposome formulations and their morphologies have been tuned formulaically,^[^ ^28^, ^29^ ^]^ several crucial aspects of RNA packaging and delivery remain poorly understood, including the dynamics of hybrid RNA‐liposome nanomaterial assembly, the mechanism of RNA loading, and the biophysical characteristics of assembled nanomaterials that interact with immune cells. Second, this formulation is a simple two‐component system generated by mixing RNAs with cationic liposomes in an aqueous solution, making it a tractable system for physics modeling with minimal parameter fitting. Lastly, the formulation components such as the cationic and ionizable lipids and RNAs are found ubiquitously in other delivery systems and therefore the methodology and findings in this study can be generalizable to a broad variety of nanotherapeutics.^[^ ^14^, ^15^, ^18^, ^19^, ^20^ ^]^

Here, we use fluorescence microscopy, quantitative image analysis, and physics modeling to track the RNA‐liposome nanoparticle assembly of our clinical cancer immunotherapy, which complexes liposomes with RNA, in real time with high specificity and throughput, across multiple length and time scales (Figure 1E). The imaging results reveal discrete steps in the nanoparticle formulation process: initially, RNA adsorbs onto single liposomes; then, RNA‐coated liposomes undergo self‐assembly through a process consistent with a Smoluchowski aggregation model, where Brownian diffusion plays a crucial role in determining the rate of cluster formation. The dynamics of this process are further influenced by RNA adsorption, which introduces a “patchy‐binding” probability between interacting clusters.

This study demonstrates the potential of using quantitative fluorescence microscopy to investigate the dynamic assembly of multi‐component biological nanomaterials at multiple scales in real time. Though several recent studies have utilized fluorescence imaging to monitor dynamics of nanoscale assemblies,^[^ ^30^, ^31^, ^32^, ^33^, ^34^ ^]^ none performed detailed mathematical modeling of these systems or fully capitalized on multicolor imaging, particularly in the context of RNA‐liposome systems. By providing model‐free, single‐particle measurements at high spatiotemporal resolution, these quantitative imaging methods can help guide the rational design of new strategies for the control and improvement of nanoparticle formulations. Such methods may be of especial importance in nanomedicine, where insight into the physicochemical properties is critical in improving therapeutic efficacy. This workflow not only facilitates optimization of RNA‐based immunotherapy and RNA vaccine development but can also be broadly applied to the study of other nanoparticle formulations when the individual components can be fluorescently labeled, such as polymer‐based or protein‐based nanoparticle engineering in drug delivery and gene therapy.

## 2 Results

### 2.1 Liposomes and mRNA Self‐Assemble into Micrometer‐Scale Structures

**Figure 2:**
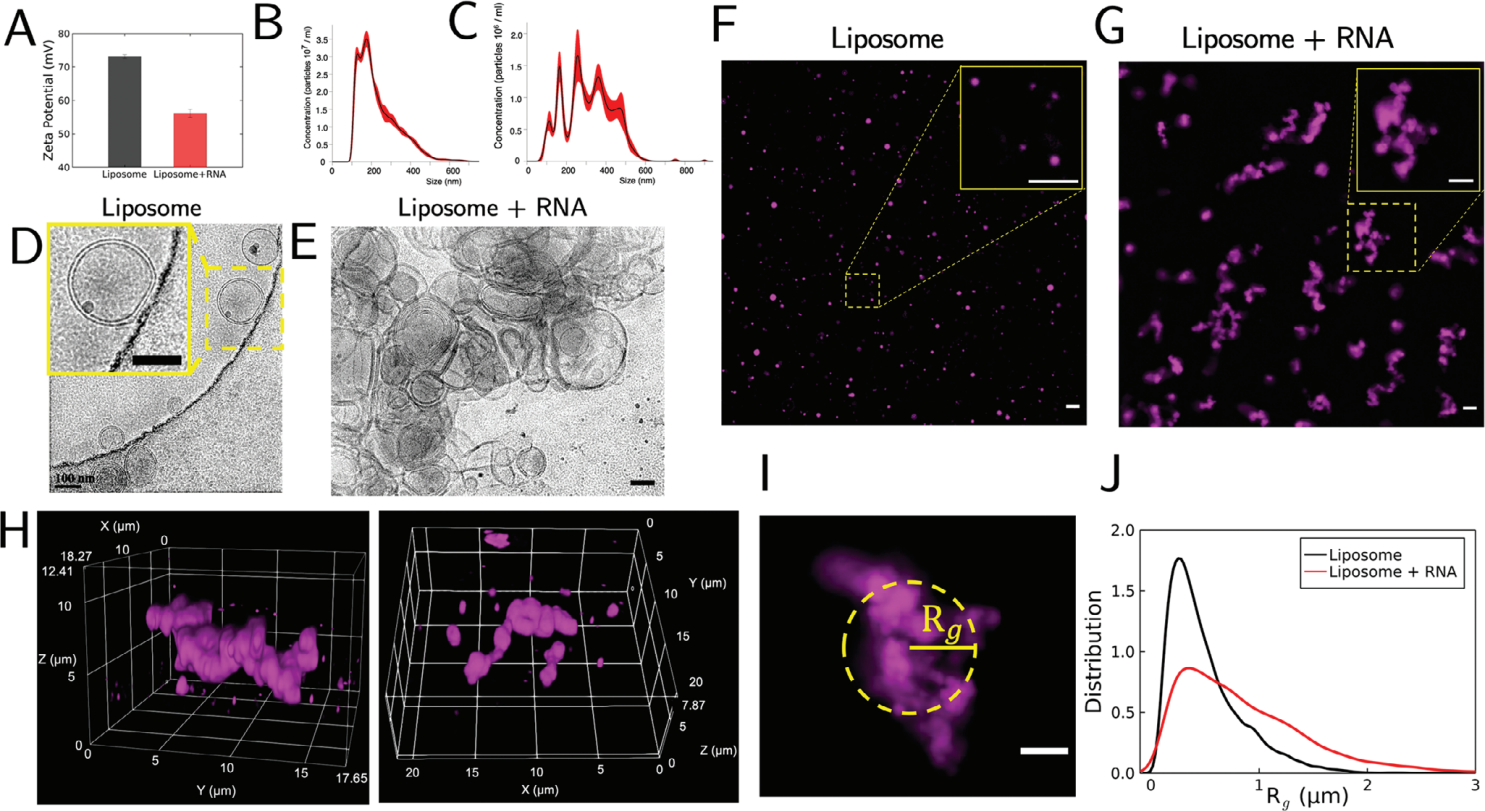
Liposomes and RNA self‐assemble into micrometer‐scale structures. A) Zeta potential measurements of liposomes with and without RNA. Data is presented as mean ± SD. (n = 5). (B) Dynamic light scattering (DLS) histogram of liposomes without RNA. Data is presented as mean ± s.e.m. (n = 5). C) Dynamic light scattering (DLS) histogram of liposomes with RNA. Data is presented as mean ± s.e.m. (n = 5). D) Electron microscopy image of liposomes without RNA. The solid yellow square in the upper left corner displays a magnified version of the region contained in the dashed yellow square. Scale bar: 100nm. E) Electron microscopy image of liposomes with RNA. Scale bar: 100 nm. Representative images showing individual liposomes in solution F) and mixing of liposomes and RNAs induce the formation of higher‐order assemblies G). Solid yellow squares in the upper right corners of each image display magnified versions of the regions contained in the dashed yellow squares in the respective images. H) Super‐resolution 3D images of liposomes with RNA. I) Representative image of an assembled liposome and RNA structure (only liposome component is shown in this image). Circle denotes its corresponding radius of gyration (R_g_), determined by customized feature‐finding algorithm, see Supporting Information for details. J) Distribution of R_g_. Number of features analyzed is N = 10 258 for liposomes and N = 7049 for liposomes with RNA. Scale bars: 5 µm.

To assess the physicochemical characteristics of the cationic liposomes composed of pure 1,2‐dioleoyl‐3‐trimethylammonium‐propane (DOTAP) with and without RNA (TriLink CleanCap EGFP, 1µg µL^−1^), we first perform standard characterization via DLS and cryo‐EM. Zeta potential measurements, in HyPure water instead of PBS, show that, as expected, the liposomes are positively charged (73.12 mV ± 0.55) and that complexation with RNA results in a drop in this surface charge (56.14 mV ± 1.19) (**Figure** **2**A). DLS (Figure 2B) and cryo‐EM (Figure 2D) data show that without RNA, liposomes are stable and fairly monodisperse; with RNA, the DLS size distribution broadens (Figure 2C), which cryo‐EM images suggest is a result of aggregation (Figure 2E).

Next, we directly visualize the morphology of the RNA‐liposome hybrid nanomaterials with fluorescence microscopy by imaging the liposomes labeled with lipid affinity dye. Importantly, DLS and cryo‐EM data suggest that the mean particle size is above diffraction limit, which must be considered prior to analysis. Before adding the RNA, the liposomes show a narrow size distribution (Figure 2F). This is consistent with prior DLS and Cryo‐EM measurements of liposomes. Strikingly, liposomes and RNA spontaneously assemble into micrometer‐scale structures of diverse sizes (Figure 2G), which qualitatively agrees with the EM images. Similar structures have been noted when complexing cationic liposomes and DNA^[^ ^35^, ^36^, ^37^ ^]^ and RNA,^[^ ^27^, ^28^, ^38^ ^]^ though morphology may depend on the exact preparation method. Interestingly, similar assembled structures are observed in the presence of serum (Figure S1, Supporting Information). Therefore, direct imaging reveals a sub‐population of assembled structures whose size regime may fall outside the detection range of conventional light scattering techniques.^[^ ^39^, ^40^ ^]^

On a closer look, many of the assembled structures show irregular, non‐compact shapes, often with elongated or branch‐like morphology. This irregular morphology was further supported by super‐resolution imaging (Figure 2H). To further quantify these structures, we performed image segmentation using standard thresholding methods and measure the radius of gyration R_g_ (Figure 2I), which is a widely used pixel‐intensity‐weighted metric to measure size of features identified in fluorescence images (see Supporting Information). Consistent with our direct observation from imaging, the R_g_ measurements clearly show that the addition of mRNA to the liposome drastically broadens the distribution of R_g_, with a long tail representing higher‐order assembly of larger micrometer‐scale structures (Figure 2J). In parallel, we calculated the fractal dimension, which is defined by how the mass of these assemblies scales with their spatial dimension.^[^ ^41^ ^]^ This quantity is a measurement of the structure's compactness and thus determines important physical properties such as density.^[^ ^42^ ^]^ We found that the fractal dimension for larger clusters agrees with branched structures in the literature,^[^ ^43^ ^]^ which corroborates the imaging observations (Figure S2, Supporting Information). The non‐circular morphology of the clusters is also seen by monitoring the circularity index, which is a number from 0 to 1 describing the degree to which segmented structures are circular (Figure S3A, Supporting Information); clusters begin highly circular and become less so over time (Figure S3B, Supporting Information).

### 2.2 RNA Initially Adsorbs onto Liposomes with RNA‐Per‐Liposome Determined by an Exponential Distribution

**Figure 3:**
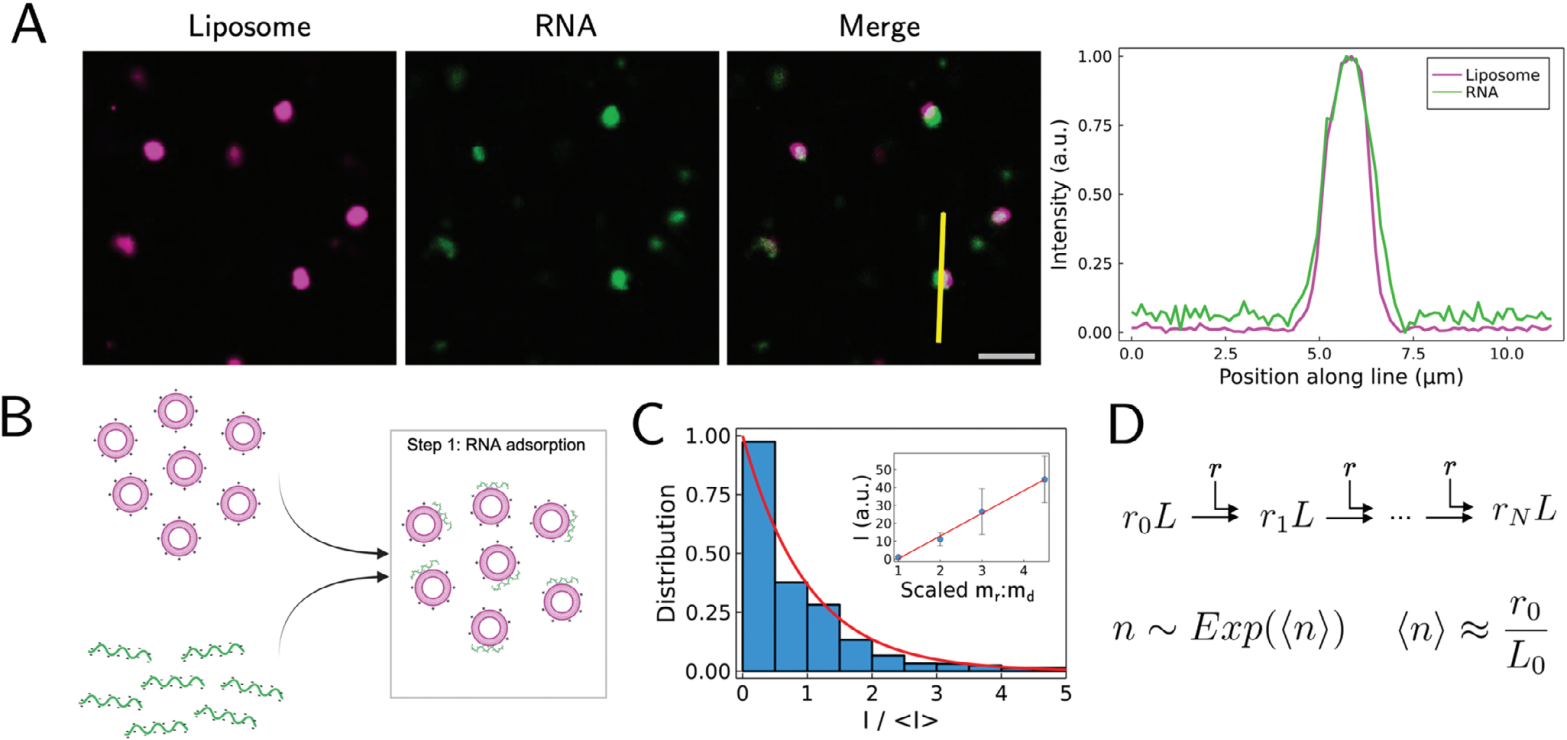
RNA initially adsorbs onto liposomes with RNA‐per‐liposome determined by an exponential distribution. A) Initially, RNA (green) adsorbs onto liposomes (magenta). Colocalization is shown in white in the Merge panel. The intensity profile corresponds to the yellow line in the merge panel. Representative images shown are acquired at the time of mixing. Scale bar: 5 µm. B) Schematic of the first stage of this system's temporal evolution following mixing. After mixing, anionic RNA (green) adsorbs onto cationic liposomes (magenta). C) Distribution of RNA intensity per liposome (I), scaled by its mean. The red curve is the theoretical prediction. Inset: the measured mean RNA intensity per liposome increases linearly with the RNA‐to‐liposome ratio, as predicted. The horizontal axis is the RNA‐to‐liposome mass ratio scaled by the mass ratio of the first data point. D) Illustration of the kinetic model used to describe the initial RNA adsorption stage (See text and Supporting Information for details). Upon equilibration, the distribution for the number of bound RNA molecules per liposome, *n*, is exponentially distributed with its mean (denoted as 〈⋅⋅⋅〉) determined by the initial RNA‐to‐liposome mass ratio. Here, *r_k_L* denotes a liposome with *k* bound RNA molecules and *r*_0_ and *L*_0_ denote the initial concentrations of RNA and liposomes, respectively.

The observation of irregular heterogeneous structures assembled by multiple liposomes prompted us to monitor the evolution of the assembly process in real time. To accomplish this, we also label RNAs with either an RNA‐affinity dye or covalent RNA‐labeling, enabling simultaneous tracking of both RNA and liposomes through two‐color imaging (**Figure** **3**A).

At the onset of RNA and liposome mixing, RNA molecules immediately adsorb onto individual liposomes, which is indicated by significant overlap seen between individual liposomes and RNAs at the time of mixing (Figure 3A). Thus, following mixing, anionic RNA adsorbs onto cationic liposomes (Figure 3B), likely driven by the electrostatic interaction between the negatively charged RNA molecules and the positively charged liposomes.^[^ ^36^, ^44^ ^]^ Using multi‐color quantitative imaging, we estimated the amount of RNA per individual liposome by measuring the RNA intensity in the fluorescence images. These measurements reveal that the data is exponentially distributed (Figure 3C). Further, the average RNA per liposome increases approximately linearly with the RNA‐to‐liposome mass ratio (Figure 3C, inset). This initial RNA adsorption step can be understood via a sequential, irreversible kinetic adsorption model (Figure 3D) (see Supporting Information). This model successfully predicts the experimental observations: the equilibrium RNA‐per‐liposome distribution is approximately exponential, and the average number of RNA molecules per liposome is proportional to the initial mass ratio of the components. Interestingly, the same result can be achieved via the maximum‐entropy principle in a model‐independent fashion (see Supporting Information). Therefore, the adsorption process indicates RNA molecules sticking irreversibly onto liposomes driven by the electrostatic interaction.

### 2.3 Higher‐Order RNA‐Nanoparticle Assembly Follows Smoluchowski Kinetics with Brownian Kernel

**Figure 4:**
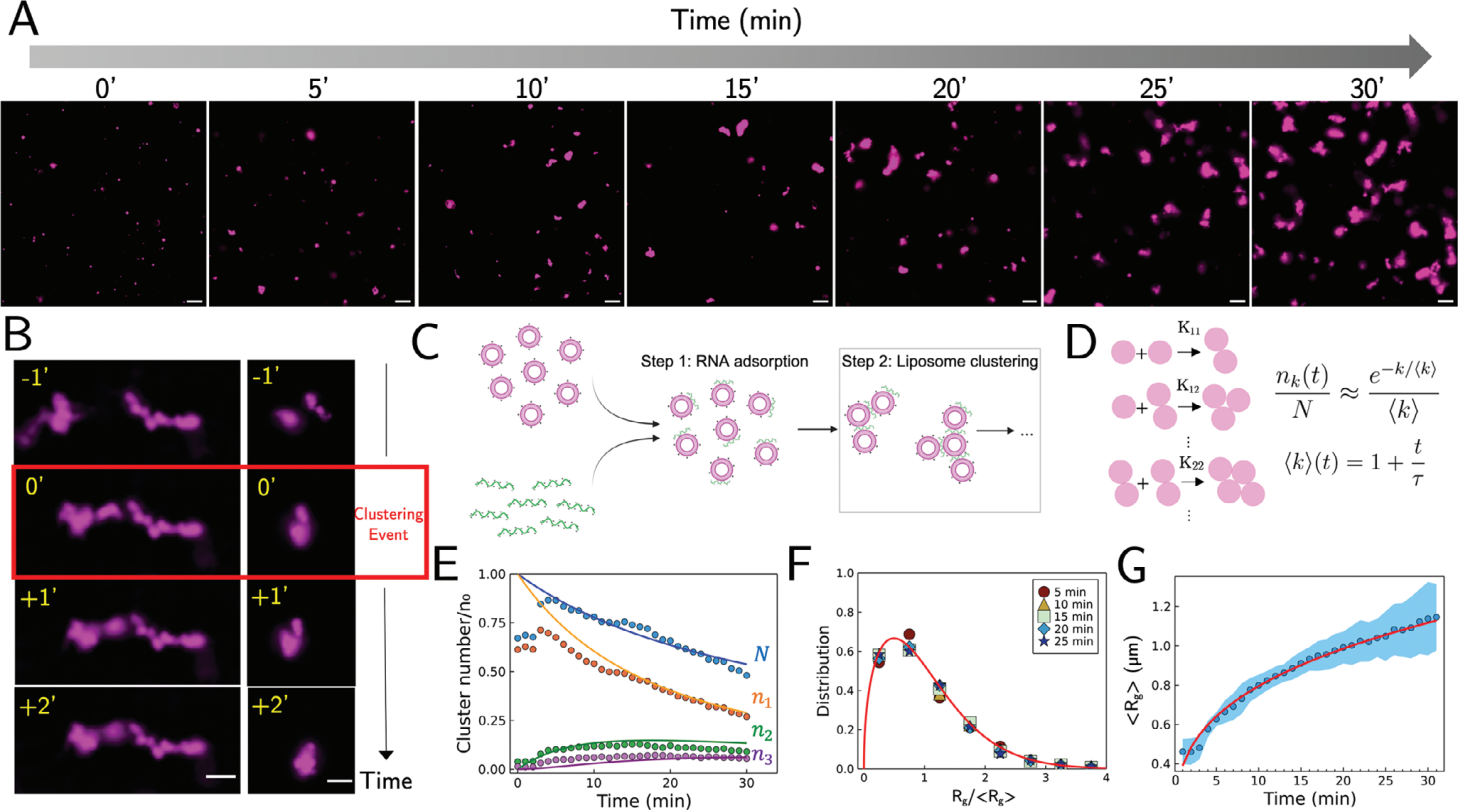
The assembly of RNA‐bound liposomes is consistent with Smoluchowski kinetics with a constant kernel. A) Real‐time images of RNA‐liposome clusters. Scale bar: 10 µm. B) Representative images of lower‐order assemblies merging into higher‐order structures upon contact. Two separately captured merging events are shown. The box in red shows the time point at which the two distinct clusters join one another irreversibly. Scale bar: 5 µm. C) Schematic of the subsequent stages of this system's temporal evolution following RNA adsorption. After the RNA adsorption stage, RNA‐coated liposomes cluster upon contact. D) Illustration of the Smoluchowski model and its approximate solution for the constant kernel, suggesting the distribution is approximately exponential (mean denoted as 〈⋅⋅⋅〉)). *K_ij_*, the kernel function, denotes the rate of particle aggregation between clusters containing *i* and *j* monomers. *n_k_* denotes the number of clusters containing *k* monomers, *N* is the total number of clusters, and τ is the growth timescale. See Supporting Information for details. E) Normalized number of clusters over time. *N* denotes total number of mRNA‐liposome clusters. *n_k_* denotes number of clusters containing *k* liposomes. Only monomer, dimer, and trimer are shown for clarity. Symbols are experimental data extracted from fluorescence images. Curves are theoretical predictions of the Smoluchowski model (see Supporting Information). F) Distribution of R_g_, scaled by its mean. This distribution is time‐invariant, as demonstrated by the collapse of multiple time points during the time evolution. The master curve is the theoretical prediction (see Supporting Information). G) Power‐law growth of the average radius of gyration (R_g_) of the assembled structures over time. Mixing happens at time 0. Data is displayed as mean ± s.t.d. The Red curve is a theoretical prediction.

Next, we sought to understand the fate of RNA‐bound liposomes following the initial stage of RNA adsorption by further time‐lapse imaging (**Figure** **4**A). We observed that RNA‐coated liposomes start to assemble by a “hit‐and‐stick” process, whereby two smaller structures make contact and stick together to form a larger assembly (Figure 4B,C). To quantitatively determine the cluster‐scale dynamics, we turn to classical Smoluchowski models of particle aggregation.^[^ ^45^, ^46^ ^]^

In Smoluchowski aggregation models, monomers in a solution assemble into higher‐order k‐mers (i.e., clusters composed of k monomers) over time, with the rate of interaction between clusters of different sizes determined by a kernel function which embeds the details specific to a system (Figure 4D). The simplest kernel capturing the essence of Brownian motion is the constant diffusive kernel, which posits that the rate of interaction between clusters is determined by diffusion and is approximately independent of cluster size (see Supporting Information). Although an approximation, this model has the benefit of having an analytical solution (Figure 4D). The underlying picture described by this kernel is that two clusters diffuse and stick to each other with a certain probability upon contact.

We image and analyze 150 000+ features corresponding to individual liposomes or assembled structures to ensure sufficient statistics to test the theoretical predictions. Remarkably, this simple physical model successfully predicts the essential features we observed in the assembly process. First, we examined the particle number within each cluster over time because such measurements are easily acquired via our imaging approach and enable quantitative comparison with the model. Our findings demonstrate that the assemblies evolve in accordance with the time‐dependent analytical solutions of the model, which describe the temporal evolution of k‐mers (Figure 4E). Second, in agreement with the model, the particles‐per‐cluster distribution is approximately exponential (see Supporting Information). Given the particles‐per‐cluster distribution, the distribution of R_g_ can be predicted via a simple change of variables (see Supporting Information), which agrees with the data (Figure 4F). Intriguingly, as predicted by the model, when scaled by their respective means, both the particles‐per‐cluster distribution (Figure S4, Supporting Information) and the R_g_ distribution (Figure 4F) are time‐invariant, which indicates that both quantities are dynamically scaled and can be sufficiently described by a time‐collapsed master curve. Third, we monitor the cluster growth as the cluster size rapidly exceeds the diffraction limit, in a size range accessible with optical imaging microscopy technique. We find that the growth of mean R_g_ over time is well‐described by a power law (Figure 4G), which is consistent with the model.

### 2.4 Cluster Growth Rate is Determined by RNA Adsorption

**Figure 5:**
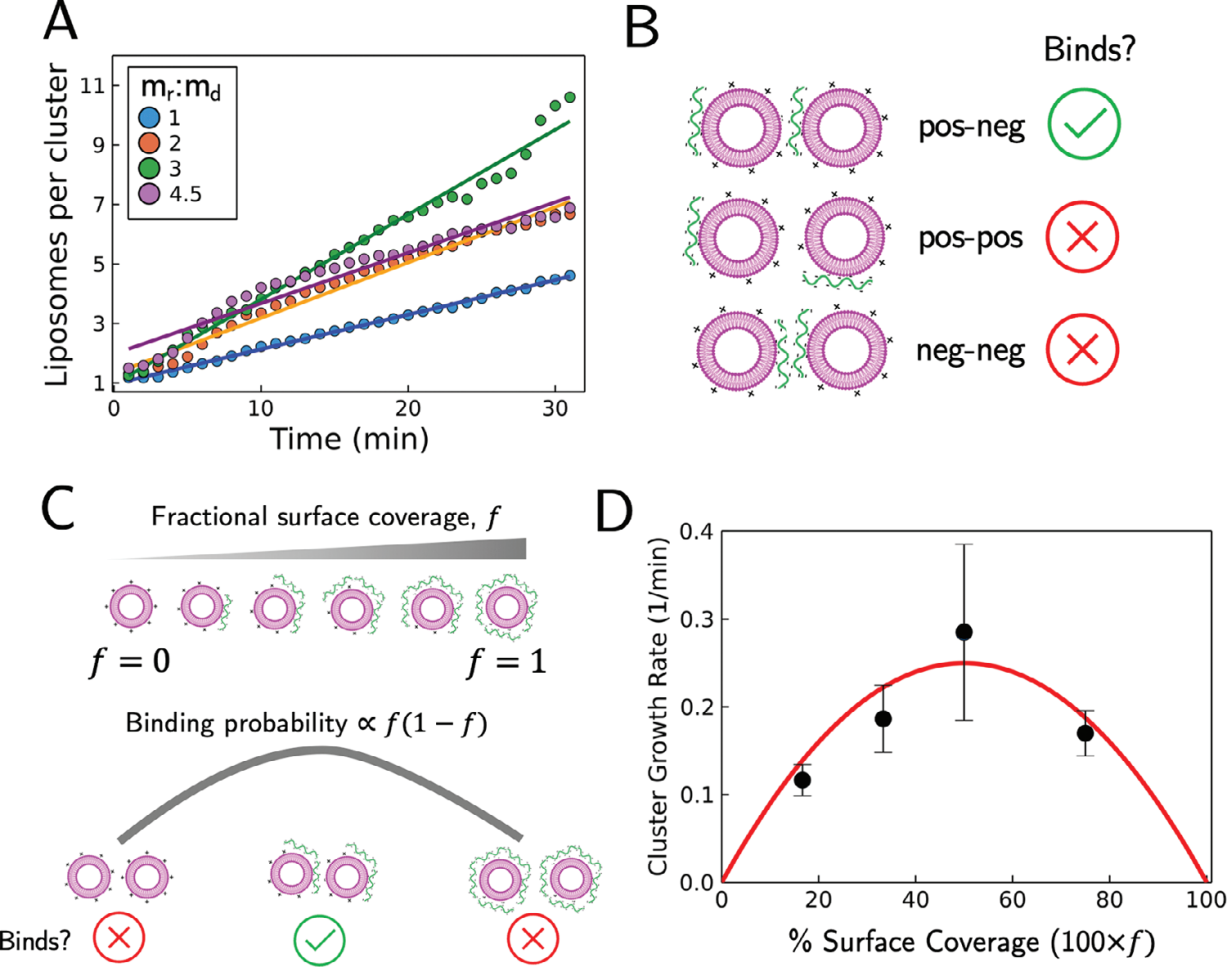
Cluster‐cluster binding probability is determined by fractional RNA surface coverage. A) Average number of liposomes per cluster versus time for different RNA‐to‐liposome mass ratios. Note that the cluster growth rate does not monotonically increase with the RNA‐to‐liposome mass ratio. Scatter points are experimental data and solid lines are linear fits to the data. B) Schematic of patchy‐binding model. Upon contact, cluster binding is favored when a positively charged liposome patch (bare, uncoated liposome surface) contacts a negatively charged RNA patch (RNA‐coated liposome surface). C) Fractional surface coverage, *f*, of RNA dictates liposome binding probability. *f*  =  0 denotes liposomes without RNA and *f*  =  1 denotes liposomes saturated with RNA. The solid gray line is an illustration of the binding probability, *f*(1 − *f*), which varies from 0 to 1. D) The dependence of cluster growth rate, which is proportional to the binding probability, on the estimated fraction of RNA coverage of liposome surface. Percent surface coverage is 100 × *f*. Data is shown as mean ± s.e.m. The red curve is a theoretical prediction (see Supporting Information).

To better understand the cluster‐cluster interaction mechanism and the dependence on RNA concentration, we studied how the system evolves from the RNA adsorption stage to the subsequent assembly stage. To accomplish this, we varied the RNA‐to‐liposome mass ratio and monitored the temporal evolution of cluster assembly. Initially, the cluster growth rate increases when increasing the RNA‐to‐liposome mass ratio. Interestingly, however, a further increase in the ratio results in a decrease in growth rate (**Figure** **5**A). This reversal suggests a critical point of RNA adsorption beyond which RNA‐coated liposome assembly is hindered.

A “patchy‐binding” model can rationalize the observation: cluster joining is only favored upon contact between oppositely charged surfaces, that is, a patch of adsorbed RNA (negatively charged) on one cluster surface and a patch of bare liposome (positively charged) on the other surface (Figure 5B); positive‐positive and negative‐negative interactions will not lead to productive formation of higher‐order clusters. Specifically, if *f*denotes the fraction of the liposome surface coated with RNA, then one would expect that the probability of binding another liposome to be approximately proportional to *f*(1 − *f*). For example, if there is no RNA in the solution and liposomes are completely free of RNA, then *f*  =  0; hence it is impossible that stable liposomes bind and *f* (1 − *f*) =  0. Similarly, if the liposomes are completely coated with RNA and *f*  =  1, then *f* (1 − *f*) =  0 (Figure 5C). Indeed, such a patchy‐binding term in the growth rate would lead to a theoretical prediction of the reversal dependence of the growth rate on the RNA‐to‐liposome ratio^[^ ^36^, ^44^ ^]^ as was seen in the data. Such a dependence of cluster growth on the adsorbed polymers has been noted in flocculation phenomena.^[^ ^45^, ^47^, ^48^ ^]^

By approximating the percent of liposome surface covered by RNA during these experiments (see Supporting Information), we see a good agreement with the theoretical prediction, which not only reproduces the overall trend of reversal in dynamics but also shows quantitative matching of cluster growth rate depending on the surface coverage (Figure 5D). These experimental observations were further validated in silico via simulated cluster assembly considering the probabilistic patchy‐binding interaction (Figure S5, Supporting Information). Thus, the negatively charged RNA acts effectively as a polymer bridge to join two positively charged liposomes during the assembly, indicating the surface coverage is an important parameter to modulate nanoparticle assembly.

### 2.5 Liposome Composition, RNA Cargo, and Solution Condition Influence Cluster Self‐Assembly Dynamics

The formulation of RNA delivery systems varies substantially based on the end goal. The liposomal composition and concomitant physicochemical characteristics, RNA cargo, and physicochemical properties of the solution in which the liposomes and RNA are mixed during complexation are subject to change.^[^ ^13^, ^17^ ^]^ Given the breadth of such changes, we use our imaging platform to gauge the impact of these various factors on RNA‐liposome cluster assembly.

**Figure 6:**
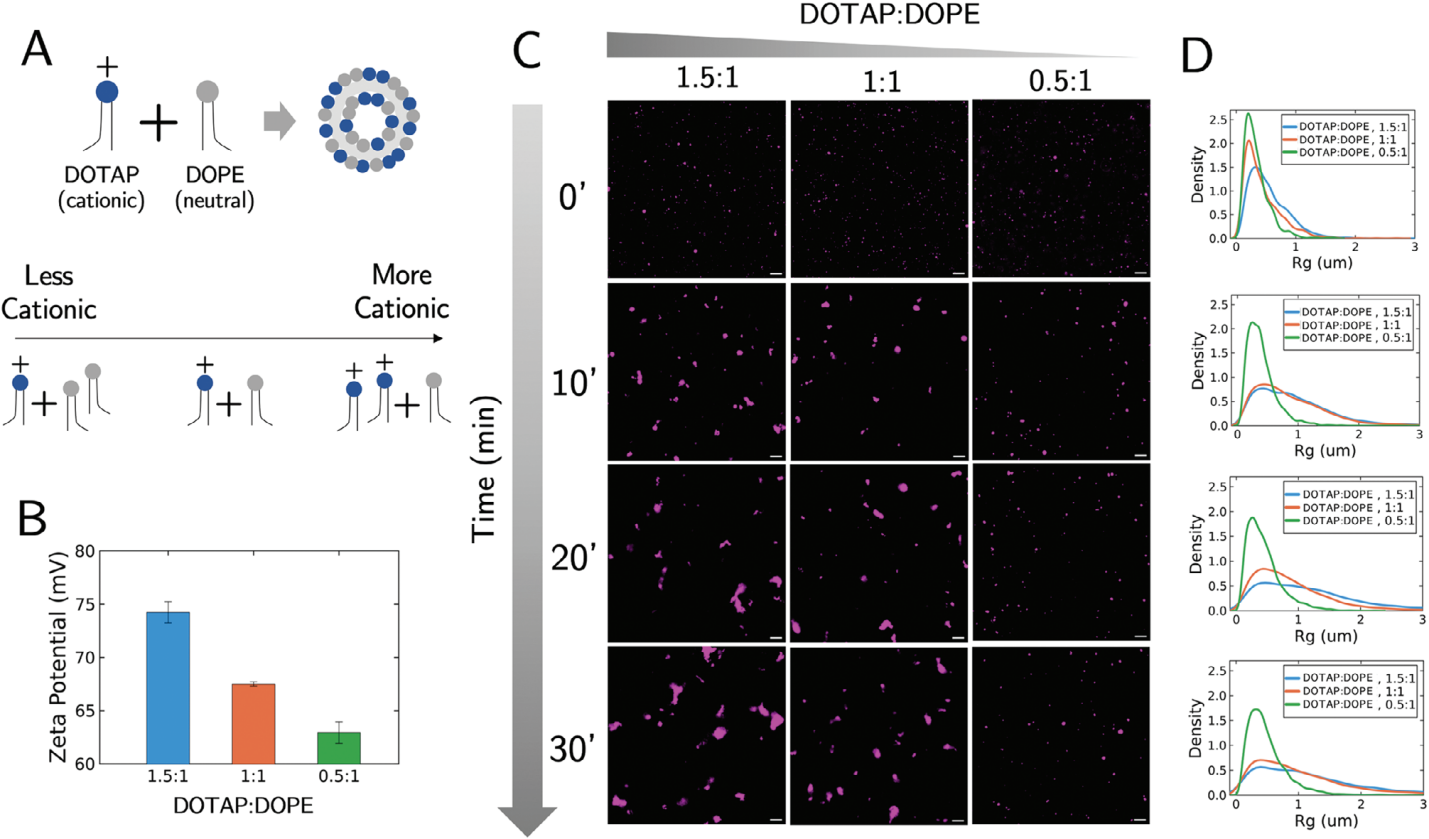
Liposome surface charge influences assembly dynamics. A) Varying the DOTAP‐to‐DOPE weight ratio (denoted DOTAP:DOPE) results in liposomes with varied degrees of positive charge. B) Decreasing DOTAP:DOPE decreases the Zeta potential of liposomes. C) Time snapshots of cluster growth as a function of DOTAP:DOPE. Scale bar: 10µm. D) Rg distribution of varying DOTAP:DOPE as a function of time (n = 3). Each plot going down the row corresponds to the timepoint to its left in C).

First, we investigated the impact of liposome surface charge and composition on self‐assembly. To accomplish this, we systematically varied the weight ratio of DOTAP to DOPE (1,2‐dioleoyl‐sn‐glycero‐3‐phosphoethanolamine), a neutral helper lipid found in many liposome‐based delivery systems.^[^ ^13^ ^]^ In principle, varying this lipid weight ratio (denoted DOTAP:DOPE, w/w), should result in liposomes with varied surface charge (**Figure** **6**A). Specifically, decreasing DOTAP:DOPE should decrease the surface charge of liposomes, which was confirmed via surface zeta potential measurements (Figure 6B). For each new liposome composition (1.5:1, 1:1, and 0.5:1 DOTAP:DOPE), we mix with the same RNA and monitor the R_g_ dynamics. The images (Figure 6C) reveal that there is a clear trend in dynamics of the R_g_ distribution with decreasing DOTAP:DOPE (Figure 6D). As DOTAP:DOPE decreases, the cluster growth rate decreases, which appears to be consistent with other studies that modify the liposome‐RNA charge ratios.^[^ ^28^, ^36^, ^37^ ^]^

**Figure 7:**
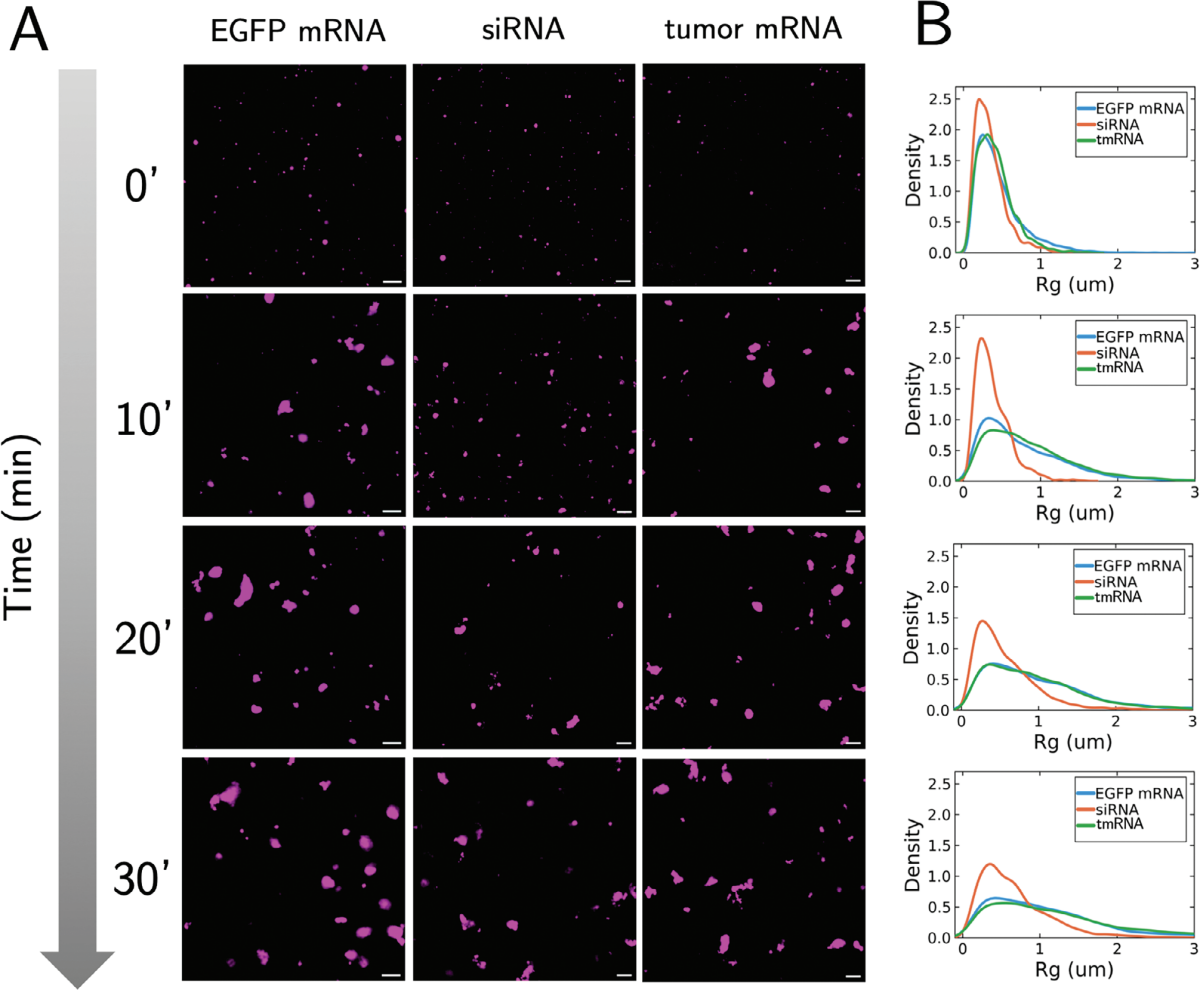
The type of RNA mixed with DOTAP influences cluster self‐assembly. A) Time snapshots of cluster growth as a function of the RNA cargo, which includes EGFP mRNA, siRNA, and tumor mRNA (tmRNA). Each column is a different RNA mixed with DOTAP, and each row is an image of the RNA‐DOTAP clusters at the specified time point. Scale bar: 10µm. B) R_g_ distribution of each RNA mixed with DOTAP as a function of time (n = 3). Each plot going down the row corresponds to the time point to its left in A).

Next, we examined the impact of RNA cargo on cluster self‐assembly. We complex three different RNAs with DOTAP: EGFP‐encoding mRNA, siRNA, and tumor mRNA (tmRNA), respectively (see Methods). Interestingly, real‐time imaging reveals that there are differences in dynamics (**Figure** **7**). The EGFP mRNA and tmRNA display similar growth dynamics (Figure 7B). However, the siRNA‐DOTAP complexes appear to grow more slowly (Figure 7B), which agrees with the previous work concerning siRNA lipoplexes.^[^ ^49^, ^50^ ^]^ Taken together, this suggests that RNA payload type should be an important formulation design consideration as it may have an impact on the cluster dynamics.

**Figure 8:**
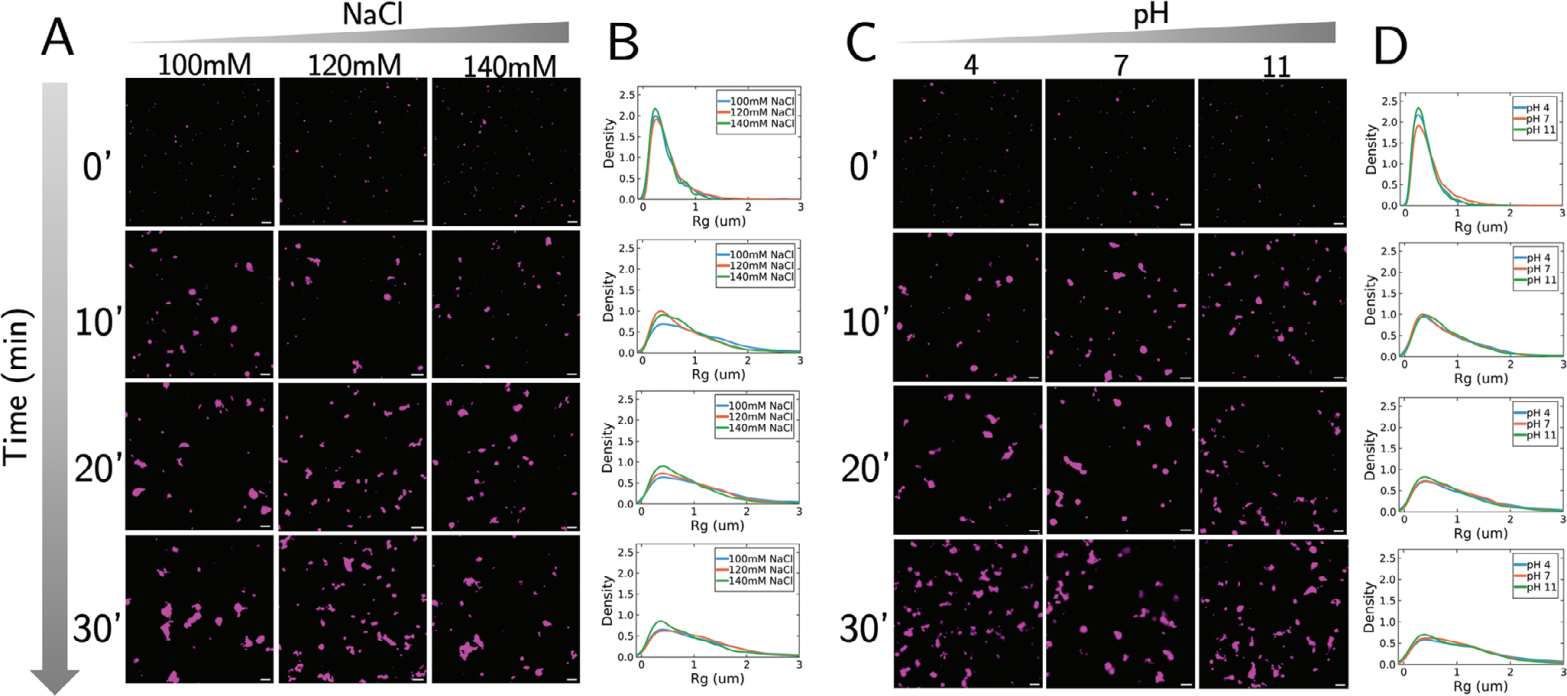
Ionic strength and pH influence cluster growth. A) Time snapshots of cluster growth as a function of the NaCl concentration. Scale bar: 10µm. B) R_g_ distribution as a function of time (n = 3). Each plot going down the row corresponds to the time point to its left in A). C) Time snapshots of cluster growth as a function of the solution pH. Scale bar: 10µm. D) R_g_ distribution as a function of time (n = 3). Each plot going down the row corresponds to the timepoint to its left in C).

Finally, we modify the solution in which the RNA and DOTAP evolve (see Methods for details). First, we modify the ionic strength of the solution via the NaCl concentration. It is well known that ionic strength impacts the aggregation of charged particles via charge screening.^[^ ^51^ ^]^ Thus, one may expect that stronger electrostatic interactions between RNA and DOTAP at lower NaCl concentrations may facilitate more rapid cluster growth. Indeed, this is what we observed (**Figure** **8**A,B). More generally, as the NaCl concentration increases, the cluster growth rate decreases, in agreement with previous studies.^[^ ^52^, ^53^ ^]^ Next, we modify the pH of the solution. Interestingly, modifying the pH does not appear to dramatically impact the cluster growth (Figure 8C,D). This may be due to DOTAP's cationic charge being robust to changes in pH.^[^ ^54^, ^55^ ^]^

## 3 Discussion

To fully harness the potential of RNA vaccines in combatting infectious diseases and advancing cancer immunotherapy, it is essential to employ assays that can dissect various stages in biomaterial self‐assembly and interactions with cells. These assays offer invaluable insights for guiding optimization efforts, enabling precise control over the physical and chemical attributes of resultant nanoparticle assemblies, including particle size, shape, and surface chemistry. These properties, in turn, exert a profound influence on cellular and tissue interactions with the nanomaterials across a spectrum of critical processes such as the efficiency of uptake by target cells,^[^ ^56^, ^57^, ^58^, ^59^, ^60^, ^61^, ^62^ ^]^ biodistribution of the nanomaterials,^[^ ^28^, ^63^, ^64^ ^]^ the escape of mRNA from endosomal vesicles into the cytoplasm for expression,^[^ ^57^, ^64^, ^65^ ^]^ and eventual immune responses.^[^ ^66^, ^67^ ^]^

This work demonstrates that direct imaging can be employed to dissect detailed assembly mechanisms of hybrid nanomaterials, thus revealing previously unrecognized opportunities to improve drug or nucleic acid payload delivery, addressing fundamental challenges in nanotherapeutics. Particles of different size ranges elicit different cellular uptake pathways and different downstream cellular processing pathways and immune responses. This work underscores the potential to address such fundamental problems via manipulation of the nanomaterial assembly process, emphasizing the critical role of understanding assembly dynamics in the rational design of nanoparticle delivery systems to fine‐tune their characteristics for optimal outcomes.

The results obtained in this study underscore the potential of fluorescence imaging to serve as a versatile tool for real‐time monitoring of the assembly of multiple components within hybrid nanomaterial systems at the single‐particle level, providing the capacity for high‐throughput data acquisition and delivering time‐resolved, component‐specific mechanistic insights that complement other nanomaterial characterization techniques. We expect that the methods and findings herein can be used to understand and control the self‐assembly of RNA‐based lipoplex formulations, which necessitates understanding both RNA adsorption and subsequent clustering. These considerations are critical for successful nucleic acid delivery and there are clear tradeoffs: larger clusters carry more RNA and are able to deliver multiple RNA payloads,^[^ ^27^ ^]^ but the cellular internalization may be adversely impacted. Mechanistic insights through correlative imaging and in vitro and in vivo assays into this self‐assembly and cellular responses may guide rational design efforts in this tradeoff. Further, given the multiplex, real‐time capabilities of fluorescence imaging, and recent development of fluorescent reporters for the various stages of mRNA delivery,^[^ ^68^, ^69^ ^]^ the approach taken in this work can be integrated into a complete fluorescent imaging workflow for any nanoparticle platform amenable to fluorescent imaging, performing upstream characterization of the delivery formulation and subsequently analyzing the cellular uptake, endosomal escape, and translation of delivered mRNA. Continuing innovation in the field of super‐resolution microscopy^[^ ^70^, ^71^ ^]^ may also open the door for this approach to be applied at even finer length scales. This work has been limited to epifluorescent imaging to provide sufficient spatiotemporal sampling for data analysis, but next‐generation imaging modalities may further improve the data acquisition capabilities herein. Further, this approach may be fruitfully combined with theoretical advances pertinent to colloidal systems^[^ ^72^, ^73^, ^74^ ^]^ and experimental approaches in vesicle dynamics^[^ ^75^, ^76^ ^]^ to expand the understanding of the dynamics of such systems. Importantly, as fluorescence dye labeling has become widely available for lipid‐, nucleic acid‐, and polymer‐based materials, this platform can be readily applied to other bio‐ and nanomaterials.

## 4 Experimental Section

### RNA‐Liposome Characterization Studies

Nanoparticle surface charge and size were determined using the Malvern Panalytical's Zetasizer Ultra and NanoSight's nanoparticle tracking analysis (NTA) equipment, respectively. Before analyzing each sample for particle size distribution and PDI using the NanoSight‘s NTA equipment, LNPs were diluted 10–25 fold with PBS and then ran through the equipment for five captures using the optimum camera settings. All captures were analyzed at the optimum threshold of detection. For surface zeta potential measurements, instead of using PBS as diluent for liposome formulation, which is standard for clinical lot formulations, we performed sample dilutions in HyPure water to prevent saline interference with zeta potential measurements. Particles were diluted 100 fold with HyClone HyPure water (Cytiva, molecular biology grade), mixed 20 times, and ran under Zetasizer Ultra (triplicate measurements) at 25 °C with 30 s incubation and plotted as average mV with standard deviation exported from the ZS explorer software. Cryo‐electron micrographs were obtained following usage of core instruments at the UF Interdisciplinary Center for Biotechnology Research.

### Liposome Generation

Raw materials for lyophilized DOTAP (1,2‐dioleoyl‐3‐trimethylammonium‐propane (chloride salt, Lot# 890890P‐500 MG‐C‐180) and DOPE (1,2‐dioleoyl‐sn‐glycero‐3‐phosphoethanolamine, lot# 850725P‐200MG‐B‐447) were obtained from Avanti Polar Lipids, now part of Croda. To develop pure liposomes and prevent ambient oxidation, we utilized a rota‐evaporator system to remove solvents and rapidly reconstituted lipids in an aqueous solution post hydration and rota‐vaporization. Briefly, chloroform was added to lyophilized DOTAP and DOPE starting material and chloroform was carefully evaporated through gentle rotation and vacuum suction before lipid film was re‐suspended in PBS for rotational water bath heating, sonication, and sterile filtration using 0.45 and 0.2 mm syringe filters.

### RNA Acquisition and Synthesis

EGFP mRNA was purchased from Trilink Biotechnologies (Cat. L‐7601). Tumor‐derived RNA was generated by extracting total RNA from the parental B16F0 cell line using Qiagen kits. Reverse transcription PCR (RT‐PCR) was then performed to create the cDNA library (Takara, Cat. 639 537 and 639 202), followed by in vitro transcription (IVT) reactions. The resulting tumor mRNA was purified using the RNeasy Kit (Qiagen, Cat. 75 162). PD‐L1 siRNA was obtained from Santa Cruz Biotechnology (Cat. sc‐39700). The quality of the mRNA was evaluated by measuring its concentration and purity with a Nanodrop spectrophotometer. Additionally, gel electrophoresis and Bioanalyzer analyses were conducted to verify the integrity and size distribution of the mRNA.

### RNA‐Liposome Preparation and Imaging

Liposomes composed of cationic 1,2‐dioleoyl‐3‐trimethylammonium‐propane (DOTAP) lipids were labeled with CellTracker CM‐DiI Dye from ThermoFisher at a final dye concentration of 1.8 µm. To make the RNA solution, 2µL of PBS was mixed with 2µg of RNA. To fluorescently label mRNA, 0.5µL of a 25x dilution (in PBS) of SYTO 9 Green Fluorescent Nucleic Acid Stain (SYTO) was then added to the RNA solution and thoroughly mixed. Proper colocalization and image calibration were ensured by colocalization analysis post‐mixing of RNA and DOTAP (Figure S6, Supporting Information). Non‐specific labeling by SYTO was ruled out by adding SYTO to DOTAP and imaging in the GFP and Cy3 channels (Figure S7, Supporting Information). To ensure there was negligible channel bleed‐through, imaging was performed in the GFP and Cy3 channels on unlabeled DOTAP with SYTO‐labeled mRNA and CM‐Dil‐labeled DOTAP with unlabeled mRNA (Figure S8, Supporting Information). Further, imaging in the brightfield channel was performed to rule out the possibility that the labeling induced particle clustering (Figure S9, Supporting Information). Potential FRET between dyes was also ruled out by measuring the total fluorescence intensity within clusters with and without RNA dye (Figure S10, Supporting Information). To prepare RNA‐liposomes for imaging, ≈ 15µg of labeled liposomes were added to an 8‐well plate (Cellvis) and image acquisition conditions were then set (See Image Acquisition Conditions). Half of the labeled RNA solution was then added, and imaging was started immediately for real‐time experiments. For initial characterization studies, imaging was performed 15 min after mixing, as this was the incubation time for the clinical formulation.

### Animal Studies

C57B/6 or Balb/c mice were i.v. Inoculated with B16F10‐OVA or K7M2, respectively, and treated with formulated RNA vaccines as previously described. Total tumor RNA was extracted from K7M2 cell lines, while tumor mRNA involved cDNA library generation from TTRNA followed by mRNA amplification as previously described.^[^ ^27^ ^]^ All experiments were approved by the University of Florida Institutional Animal Care and Use Committee (IACUC #202009685).

### Image Acquisition Conditions

Imaging was performed using a Nikon Eclipse Ti2‐E and X‐Cite XLED1 Multi‐Triggering LED Illumination System. The microscope was equipped with an ASI piezo stage to achieve fast automated imaging acquisition in three dimensions. Super‐resolution images in Figure 2H of the main text were acquired with a Nikon NSPARC. Automated image acquisition was controlled by Micro‐Manager.^[^ ^77^ ^]^ Imaging was performed in the GFP and Cy3 channels (both at 20% full illumination intensity at exposure times of 20 ms) over a period of 30 min in 1‐minute intervals, with 3×3 tiling (tiles separated by 300 µm) and z‐stacks at 0 µm (bottom of well), 25 µm, 50 µm, 75 µm, and 100 µm.

### Image Segmentation and Analysis

Image segmentation with custom scripts was carried out entirely in the open‐source image analysis software, FIJI (1.5.3q).^[^ ^78^ ^]^ Cy3 images were first preprocessed using a morphological top‐hat filter and then a Gaussian filter to reduce background and noise. Automatic thresholding and segmentation were then performed (Figure S11, Supporting Information). Identified features were then overlaid with the original images to take intensity measurements. GFP channel analysis was performed by first preprocessing by using a rolling ball to perform background subtraction and then filtered using a Gaussian filter, and then overlaying the segmented Cy3 images and measuring. To estimate the number of particles per cluster, the average intensity of single liposomes without RNA was first estimated; the estimated number of particles per cluster was then the total fluorescent intensity of the cluster divided by the average single liposome intensity. All measurement results (as.csv files) were loaded into the open‐source programming language Julia (v1.7) to perform all analyses and generate all plots.

### Modification of RNA‐NP solution

To modify the NaCl concentration, the PBS in the RNA mixture was replaced with either deionized water or a 10X PBS solution (NaCl ≈1370mm) diluted with deionized water, bringing the final NaCl concentration to 100 and 140mm, respectively. To modify the pH, an HCl solution with pH of 1 or a NaOH solution with pH of 14 was added to the RNA‐liposome mixture until the pH read 4.0 and 11.0 on an Apera PH700 pH meter. Imaging was carried out in all modified conditions as before.

## Conflict of Interest

E.J.S. is a paid consultant for Siren Biotechnology and Nature's Toolbox, Inc. (NTx). The manuscript discusses patented technologies from H.M.G, A.G., and E.J.S that are under license by iOncologi, Inc. All other authors declare no competing interests.

## Author Contributions

M.C.C., J.G., E.J.S., and H.M‐G. conceived and designed the study; M.C.C., D.S., R.M., J.A., B.S., and A.G carried out experiments; M.C.C. performed analysis; M.C.C. and A.Z. performed simulations; M.C.C. and J.G. wrote the manuscript. All authors contributed to reviewing and editing of the manuscript.

## Data, Materials, and Software

All data needed to evaluate the conclusions in the paper are present in the paper and/or the Supporting Information.

## Supporting information



Supporting Information

## Data Availability

The data that support the findings of this study are available in the supplementary material of this article.
