# Supplementary material for: Multi‐Step Assembly of an RNA‐Liposome Nanoparticle Formulation Revealed by Real‐Time, Single‐Particle Quantitative Imaging
Source: Adv Sci (Weinh). 2025 Jan 31;12(12):2414305. doi: 10.1002/advs.202414305 (PMC11948016; doi:10.1002/advs.202414305)
Supplement: Supplementary file 1 — Supporting Information [file ADVS-12-2414305-s001.pdf]

ADVANCED SCIENCE
Open Access

Supporting Information

for *Adv. Sci.*, DOI 10.1002/advs.202414305

Multi-Step Assembly of an RNA-Liposome Nanoparticle Formulation Revealed by Real-Time, Single-Particle Quantitative Imaging

Michael C. Chung, Hector R. Mendez-Gomez, Dhruvkumar Soni, Reagan McGinley, Alen Zacharia, Jewel Ashbrook, Brian Stover, Adam J. Grippin, Elias J. Sayour and Juan Guan**

Supporting Information for

Multi-step assembly of an RNA-liposome nanoparticle formulation revealed by real-time, single-particle quantitative imaging

Michael C. Chung[1,2], Hector Mendez-Gomez[3], Dhruvkumar Soni[3], Reagan McGinley[4], Alen Zacharia[2], Jewel Ashbrook[5], Brian Stover[6], Adam J. Grippin[7], Elias J. Sayour[3,6,*], and Juan Guan[1,*]

1. University of Texas at Austin, Division of Chemical Biology and Medicinal Chemistry, College of Pharmacy, Austin, TX 78712, United States
2. University of Florida Department of Physics, Gainesville, FL 32611, United States
3. University of Florida Lillian S. Wells Department of Neurosurgery, Preston A. Wells, Jr. Center for Brain Tumor Therapy, Gainesville, FL, 32610 , United States
4. University of Florida Department of Microbiology and Cell Science, Gainesville, FL 32603, United States
5. Middlebury College Department of Physics, McCardell Bicentennial Hall, Middlebury, VT 05753, United States
6. University of Florida Department of Pediatrics, Division of Pediatric Hematology Oncology, Gainesville, FL, 32610, United States
7. University of Texas MD Anderson Cancer Center, Department of Radiation Oncology, Houston, TX, 77030, United States

Supporting Information Text

Image Analysis

Radius of Gyration. The radius of gyration is an size metric that naturally arises in scattering theory. In the context of image analysis, instead of weighting positions by mass, we use the following intensity-weighted metric:

$$R_g^2 = \frac{\sum_{i=1}^{N} I_i(\mathbf{r}_i - \mathbf{r}_c)^2}{\sum_{i=1}^{N} I_i}$$

Where I_i is the intensity of pixel i in the image, $\mathbf{r}_i$ is the spatial position of pixel i, N is the total number of pixels in the image, and $\mathbf{r}_c$ is the spatial position of the 'center of intensity', the analog of center of mass, $\mathbf{r}_c = \sum_{i=1}^{N} I_i \mathbf{r}_i / \sum_{i=1}^{N} I_i$.

mRNA Adsorption Model

Kinetic Model. Let $[r]$ and $[r_k D]$ denotes the concentration of free (unbound) mRNA and liposomes bound with k mRNA molecules, respectively. Further, let r_0 and D_0 denote the initial concentration of mRNA and liposomes, respectively. Assuming that the mass of each component is conserved (neglible degradation), then we have the following conservation laws:

$$\sum_{k=0}^{\infty} [r_k D] = D_0 \tag{1}$$

$$[r] + \sum_{k=0}^{\infty} k[r_k D] = r_0 \tag{2}$$

The first equation is liposome conservation and the second is mRNA conservation. Now, for all k, we assume the system evolves according to the following irreversible reaction:

$$[r] + [r_k D] \longrightarrow [r_{k+1} D]$$

The accompanying ODEs:

$$\frac{d[r_0 D]}{dt} = -k_f^{(0)}[r][r_0 D], \quad k = 0 \tag{3}$$

$$\frac{d[r_k D]}{dt} = -k_f^{(k)}[r][r_k D] + k_f^{(k-1)}[r][r_{k-1} D], \quad k > 0 \tag{4}$$

Where $k_f^{(k)}$ is the rate at which the forward reactions proceed. Assuming that these rates are approximately independent of the number of bound mRNA on each liposome, we use an exponential ansatz of the form $[r_k D] = A(t)a(t)^k$. Inserting the ansatz into the liposome conservation law (Eqn. 1):

$$A = D_0(1 - a) \tag{5}$$

Inserting the ansatz into the conservation law for mRNA (Eqn. 2) and using Eqn. 5:

$$r = r_0 - D_0 \frac{a}{1 - a} \tag{6}$$

Now we insert this into Eqn. 3 and simplifying:

$$\frac{da}{dt} = -(r_0 + D_0)a + r_0 \tag{7}$$

In equilibrium:

$$a = \frac{r_0}{r_0 + D_0} \tag{8}$$

Thus, the distribution is

$$\frac{[r_n D]}{D_0} = \left(1 - \frac{D_0}{r_0 + D_0}\right)^n \left(\frac{D_0}{r_0 + D_0}\right) \tag{9}$$

$$\approx \frac{1}{\bar{n}} e^{-n/\bar{n}}, \quad \bar{n} = 1 + \frac{r_0}{D_0} \tag{10}$$

Thus, the distribution is exponential and determined by the mRNA:liposome ratio.

Max Entropy. Interestingly, the distribution can also be derived in a completely model-independent way using the principle of Max Entropy. Classically, this formalism has its roots in the works of Boltzmann [1] and Gibbs [2], where it was used to connect macrostates to microstates in statistical mechanics. In the age of information theory, Jaynes and others have used it as a method for making unbiased inferences of probability distributions given constraints [3]. For our purposes, the constraints are simple: conservation of mRNA and liposomes. Assuming that the free mRNA is zero at equilibrium and letting $p_k = [r_k D]/D_0$, our equation to optimize is:

$$-\sum_{k=0}^{\infty} p_k \log(p_k) + \alpha \left(\sum_{k=0}^{\infty} p_k - 1 \right) + \beta \left(\sum_{k=0}^{\infty} k p_k - \frac{r_0}{D_0} \right) \quad [11]$$

The first constraint is a rewritten form of liposome conservation and the second is mRNA conservation, with α, β being Lagrange multipliers. Optimizing this function with respect to p_k, one can recover the result of the previous subsection, Eqn. 10.

The Smoluchowski Model

Introduction. As described in the main text, the Smoluchowski model is an infinite set of nonlinear ordinary differential equations that describe the temporal evolution of clusters [4]. The temporal evolution is dictated by a kernel function that describes the rate of cluster growth, which is generally a function of the sizes of the interacting clusters. This class of equations make several approximations: (1) they are mean-field equations, (2) interactions of more than two bodies are neglected, and (3) system is large enough that a continuous approximations for particle concentrations is adequate. This being said, for discrete particles, the form of these equations is:

$$\frac{dn_k}{dt} = \frac{1}{2} \sum_{i+j=k} K_{ij} n_i n_j - n_k \sum_{i=1}^{\infty} K_{ik} n_i \quad [12]$$

With K_{ij} being the kernel and n_k being the concentration of clusters of size k. The first term on the right-hand side is the rate at which n_k are created via aggregation and the second term is the rate at which these clusters are lost via aggregation with clusters of other size.

Maximum Likelihood Probability Distribution (MPD). Many efforts have be devoted to simplifying analyses of the Smoluchowski equations via a MPD approach [4]. Particularly of note, Matsoukas has recently used principles from thermodynamics and statistical physics to build a general framework for relating a kernel to its MPD [5]. In this framework, without derivation, the MPD (denoted by an asterisk) for this class of equations is:

$$\frac{n_k^*}{N} = w_k \frac{e^{-\beta k}}{q} \quad [13]$$

Where N is the total particle number and w_k, β, q are terms ultimately determined by the kernel function. It should be noted that this formalism can be used to derive all known analytical solutions of the Smoluchowski equations.

Transforming from cluster distribution to size distribution. The cluster distribution (probability density function) for the constant kernel is an exponential distribution: $p(x) = e^{-x/\bar{x}}/\bar{x}$, where x is the cluster size. Given this, we can derive the size distribution using the relation $r = Ax^{1/d_f}$, where A and d_f are constants and r is the cluster radius. To do so, we can use standard change-of-variable techniques in probability theory [6] and get:

$$p(r) = \frac{d_f}{\bar{r}} \left(\frac{r}{\bar{r}} \right)^{d_f - 1} e^{-(r/\bar{r})^{d_f}}$$

Where $\bar{r} = A\bar{x}^{1/d_f}$.

The Constant Brownian Kernel. The simplest possible choice of kernel is the constant kernel. Originally, this kernel was used by Smoluchowski as an approximation for a complete diffusive kernel to get an analytically-tractable solution of how clusters evolve under Brownian motion [7]. Assuming we have a monodisperse solution of particles at $t = 0$, the time-dependent analytical solution to these equations (Eqn. 12) under this choice of kernel is:

$$\frac{n_k(t)}{n_0} = \frac{(t/\tau)^{k-1}}{(1 + t/\tau)^{k+1}} \quad [14]$$

Where τ is the timescale for aggregation and is proportional to the inverse of the constant kernel value; n_0 is the initial concentration of monodisperse particles. Upon summation, one can also derive the total cluster number:

$$N = n_1(t) + n_2(t) + ... + n_k(t) + ... = \frac{n_0}{1 + t/\tau} \quad [15]$$

In Fig. 4D of the main text, the solid curves were estimated by scaling by n , which was estimated as the N(0), and then fitting N to estimate τ; this τ was then used to generate all other n_k curves.

In addition to the explicit time-dependent solutions, one can also derive the resulting cluster distribution, which is naturally related to the time-dependent solution above. Using MPD or other approaches, it can be shown that the cluster distribution in the continuous limit is exponentially distributed. For the case of the constant Brownian kernel, $\bar{x} = \bar{x}(t) = 1 + t/\tau$ and the substitution of this into the cluster distribution can recover the time-dependent solution above. An interesting feature of this model and the MPD theory is that it predicts that the time-dependence of the cluster distribution is contained in the mean of the distribution, implying that scaling the distribution by its mean should result in a time-independent distribution.

The Impact of Polymer Bridging and Estimation of the Percent Surface Adsorption. Given the hypothesis that RNA adsorption onto liposomes is facilitating cluster growth due to an attractive electrostatic interaction, an intuitive possible model of this phenomenon is that RNA is serving a 'sticky patch' on the liposome surface that can serve as an area binding other liposomes on areas without bound RNA. This physical picture then introduces a probabilistic binding term into the kernel. Specifically, if θ is the fractional surface coverage of bound RNA on the liposome surface, then the probability of binding between two particles is proportional to $\theta(1-\theta)$. This is simply a mathematical expression of the fact that, within the scope of this model of RNA-mediated growth, that a 'patch' of RNA must meet a bare area of liposome to favor clusters sticking.

To estimate the percent surface adsorption in Fig. 5C of the main text, we assume that the fractional surface coverage θ is linearly related to the RNA-to-liposome mass ratio, $\frac{m_r}{m_d}$ (i.e. $\theta = \alpha\frac{m_r}{m_d}$). To then estimate the fractional surface coverage, we fit the growth rate to the function:

$$\beta\left(\alpha\frac{m_r}{m_d}\right)\left(1-\left(\alpha\frac{m_r}{m_d}\right)\right)$$

In doing so, we get estimates of α, β. The percent surface coverage is then $100\times\theta$.

Simulation

Agent-based simulation of 'patchy' binding kinetics. A simulation of liposome aggregation was conducted using an agent-based model. The simulation was implemented using the Agents.jl package [8] as a backend in Julia version 1.8.5. Liposomes were modeled as circular objects with the RNA-coated region stored as the endpoints of an arc. These liposomes drift and interact with one another in a two-dimensional space with periodic boundary conditions. Binding occurs between two liposomes if their point of interaction lies on the surface of only one liposome's RNA-coated region. The resulting bound clusters of liposomes move and rotate as one object.

The simulation was divided into discrete time steps, set to the damping time of the momentum of a single liposome in Brownian motion. The Langevin equation was used to model the motion of liposomes and clusters, and was numerically solved by discretizing using the Euler-Maruyama method [9]. The rotational diffusion of each particle was simulated by randomly drawing an angle at each time step from the following probability density distribution [10].

Data on the number of liposomes and radius of gyration of clusters was recorded every 50 time-steps. We varied the mRNA angular coverage of liposomes and performed 20 independent runs of 1000 steps to compare with our experimental results. The growth rate was determined by fitting a line to the time versus average cluster growth curve. All code and data can be found at https://github.com/Alz314/liposome-aggregation.

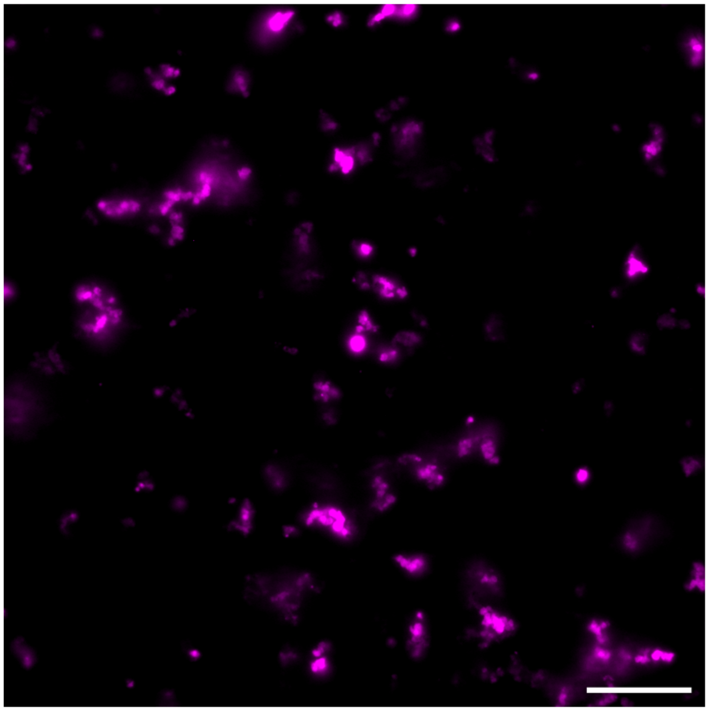

Figure S1. Representative image of RNA-liposome nanoparticles aggregates in serum. Both images shown consist of nanoparticles that were prepared and subsequent mixed with equal volumes of fetal bovine serum and imaged after 15 minutes. Scale bar: 20µm.

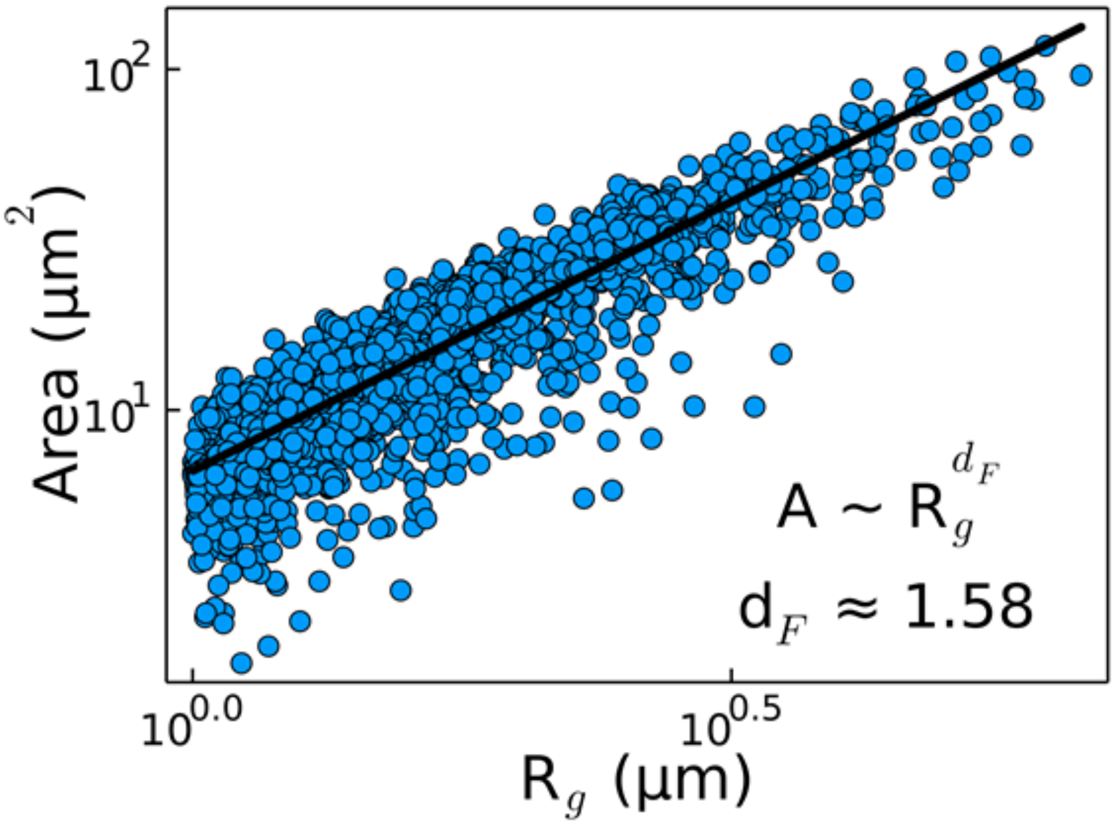

Figure S2. Log-log of area of identified cluster plotted against R_g. Exponent extracted from fit is the fractal dimension (d_F).

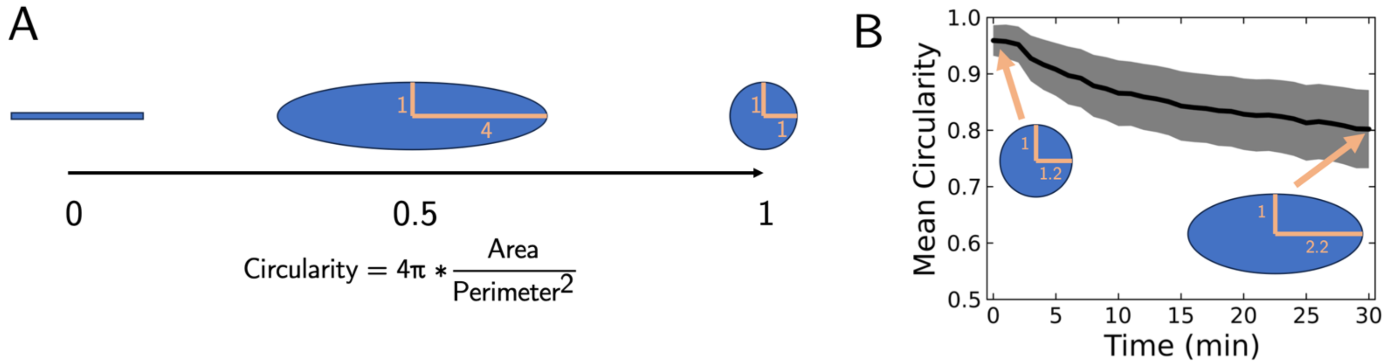

Figure S3. RNA-liposome clusters are non-circular. (A) An illustration of the circularity index, which, as its name suggests, measures the circularity of a segmented object. A line has a circularity of 0, while a circle has a circularity of 1. An ellipse with an aspect ratio (AR) of 4 has a circularity of approximately 0.5. (B) RNA-liposomes begin highly circular and become less so over time. On the figure, we visualize the degree of circularity by depicting an ellipse with as AR corresponding to the circularity shown.

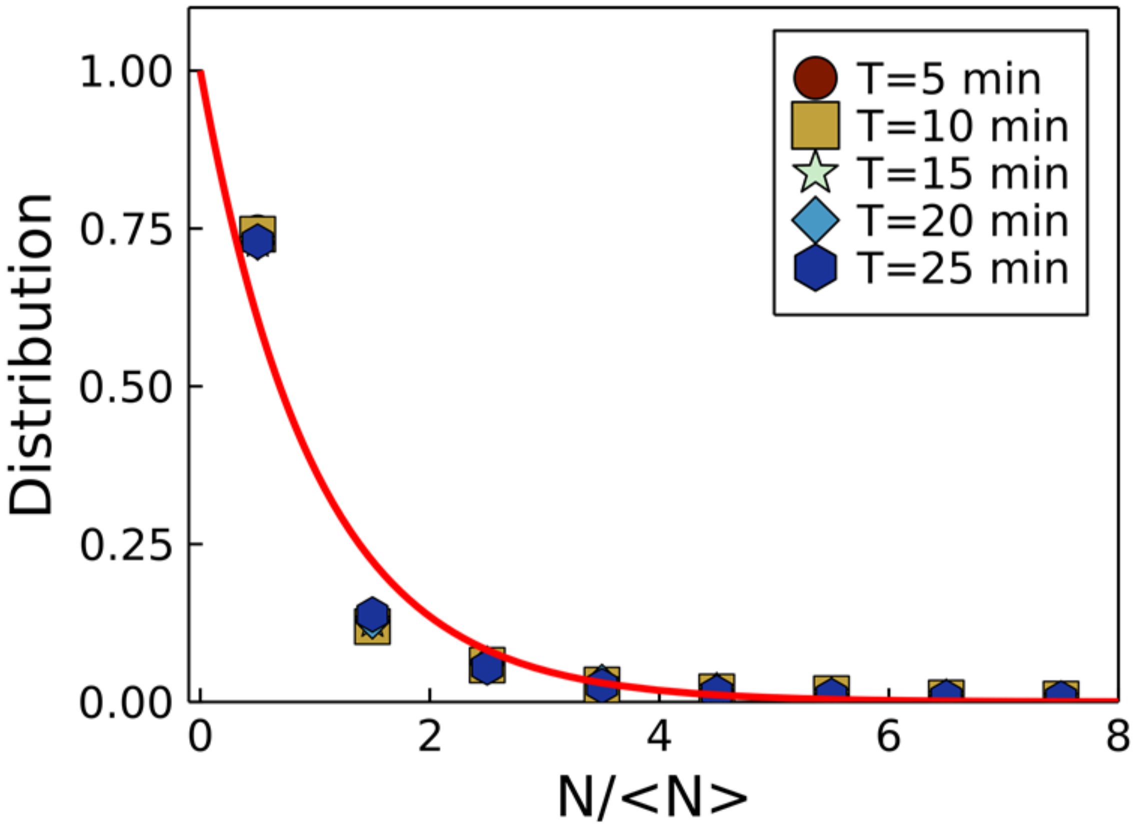

Figure S4. Distribution of particles per cluster scaled by its mean. This distribution is time-invariant, as demonstrated by plotting multiple timepoints during the system's evolution. Red curve is the theoretical prediction.

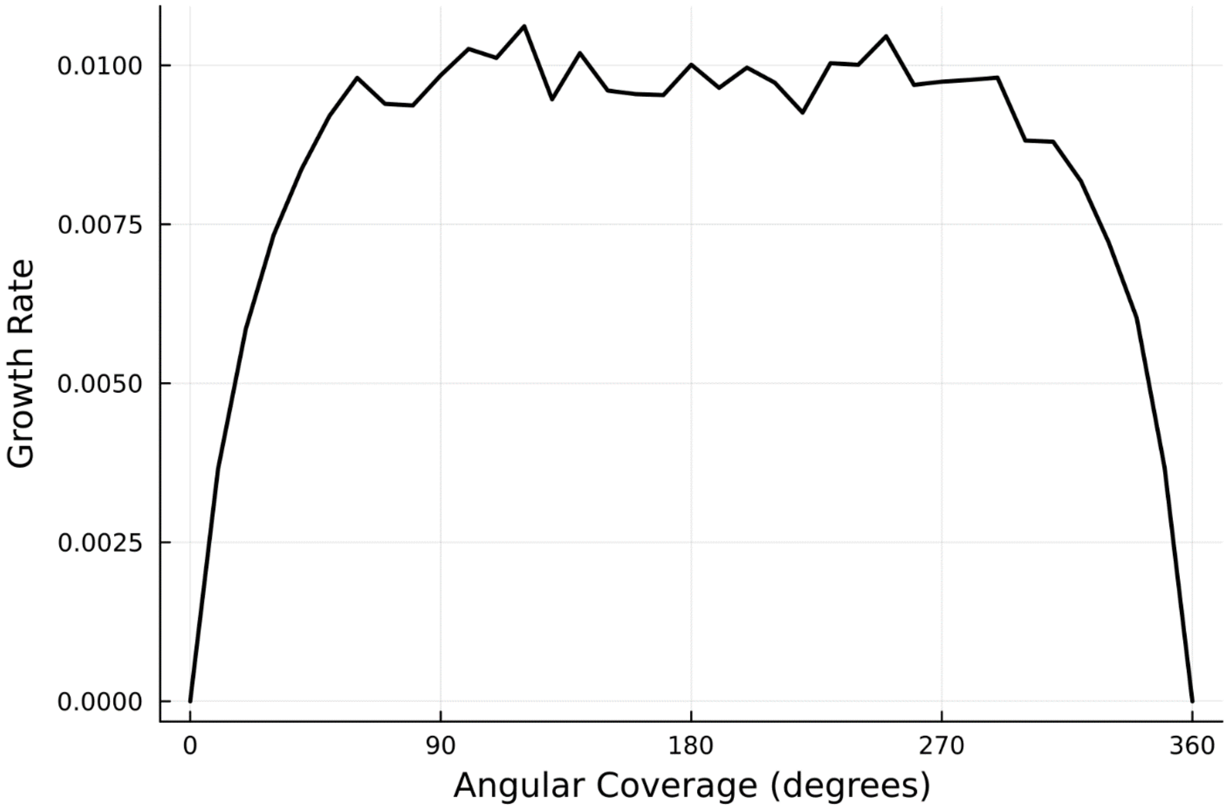

Figure S5. Simulated cluster growth rate vs. total angular coverage of RNA on liposomes (See Supplementary Methods for details).

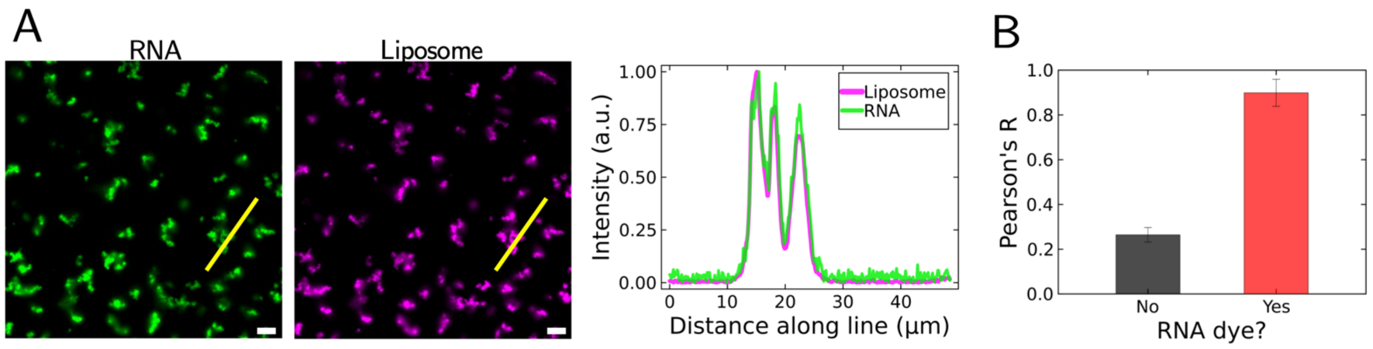

Figure S6. Intensity of RNA dye (imaged in GFP) colocalizes with intensity from liposome dye (imaged in Cy3). (A) Two-color image post-complexation of stationary clusters. Example plot of intensity along the yellow line shows high spatial colocalization. Scale bar: 10μm. (B) Two-color images are highly colocalized, as measured by Pearson's R coefficient, which measures correlation in pixel intensities from the two channels (n=9). The gray (red) bar displays the Pearson's R coefficient in the GFP and Cy3 channels without (with) the addition of RNA dye. Data is presented as mean ± std.

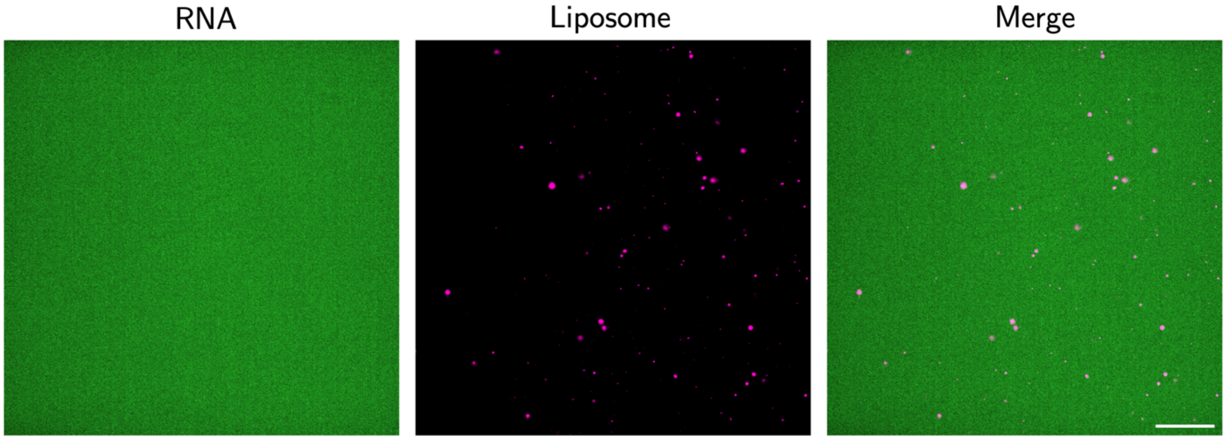

Figure S7. RNA labeling is specific. RNA dye mixed with labeled liposomes (Cy3) reveals that no structures are detected in the GFP channel. Scale bar: 20µm.

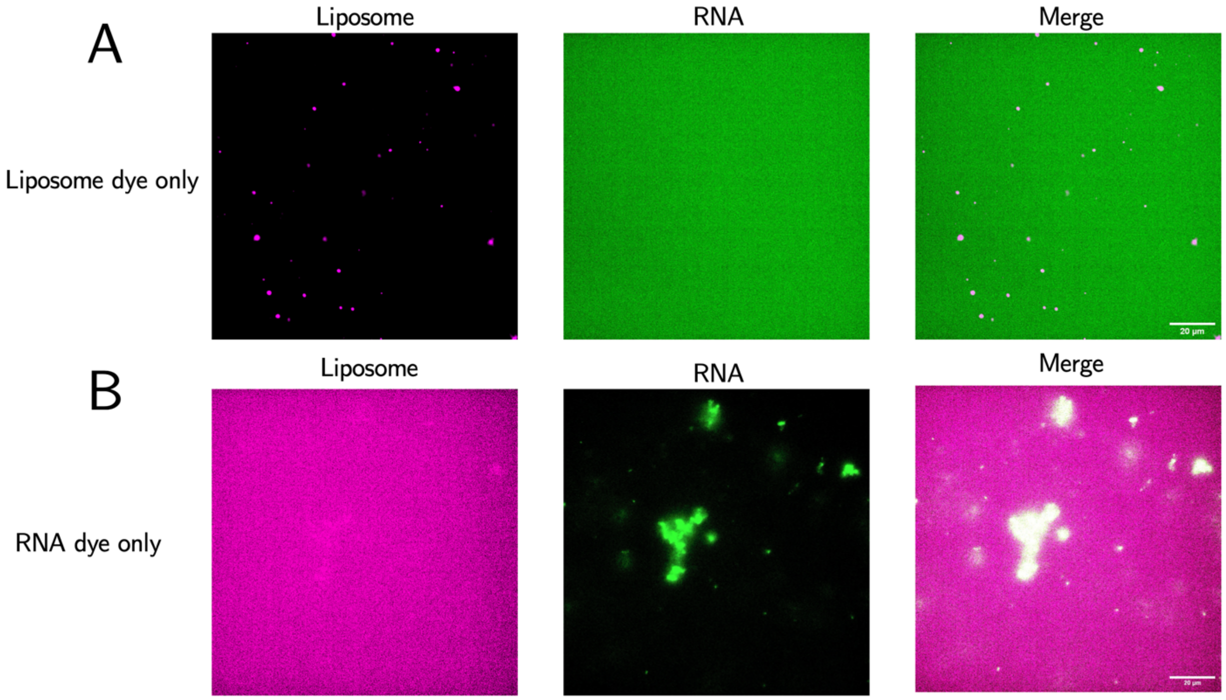

Figure S8. Measurement of overlap across Cy3 (Liposome) and GFP (RNA) channels. (A) Liposomes labeled with liposome dye (Cy3) only. No bleed-through seen in GFP channel. (B) RNA-liposome clusters labeled only with RNA dye (GFP). No bleed-through seen in the Cy3 channel. Scale bar for all images is 20 µm.

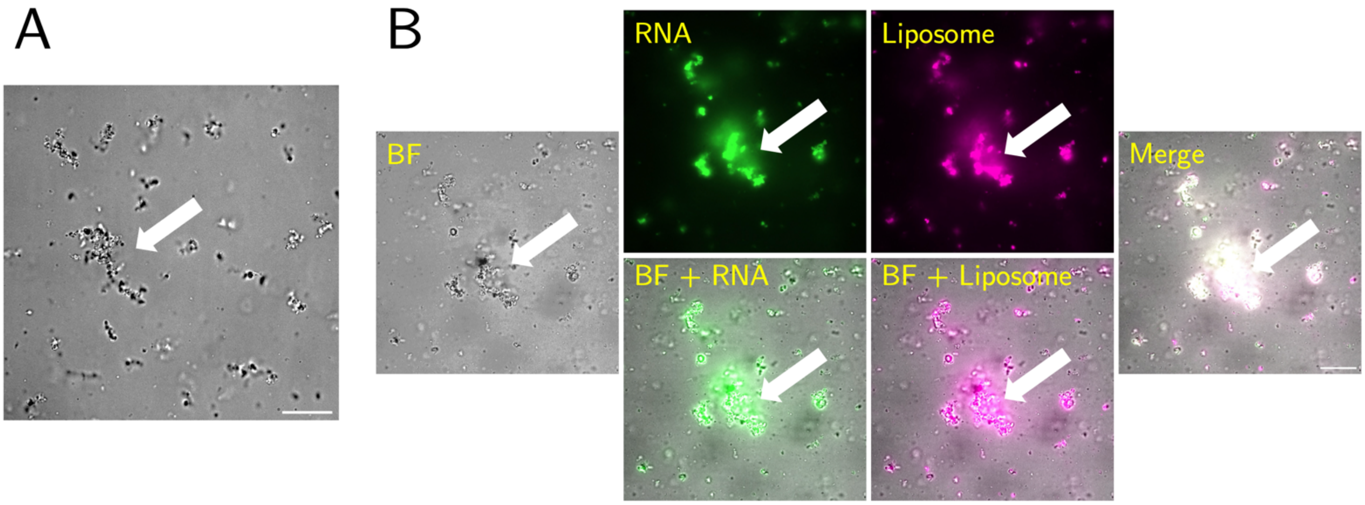

Figure S9. Fluorescent labels do not induce aggregation of RNA-liposomes. (A) Brightfield image of RNA-liposomes prepared without any fluorescent dyes. Arrow indicates an example of an aggregated structure. Scale bar: 20 µm. (B) Images in multiple channels demonstrating aggregates seen in the brightfield (BF) channel colocalize with component specific dyes. RNA is labeled in GFP channel and liposomes are labeled in Cy3 channel. Scale bar: 20 µm.

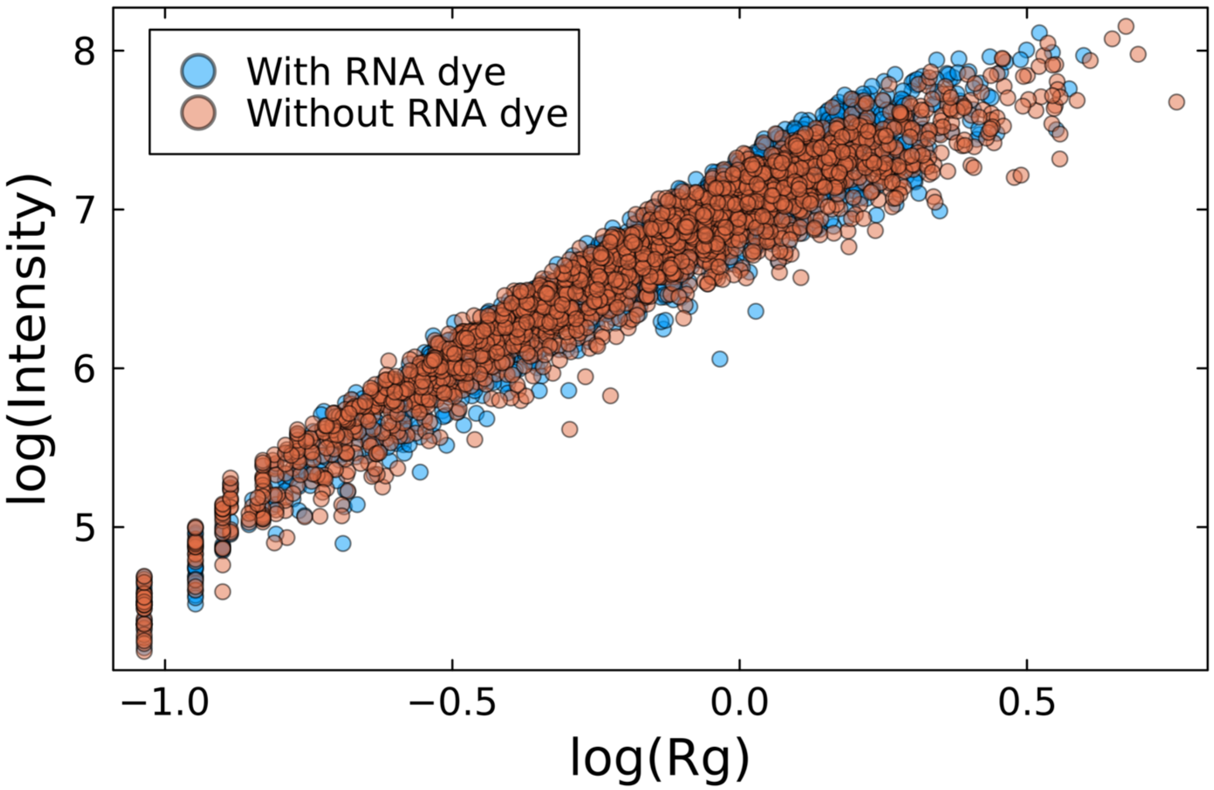

Figure S10. The presence of RNA dye (imaged in GFP) does not impact the liposome intensity (imaged in Cy3). The fluorescence intensity was measured for clusters of particles with and without RNA dye. The scaling of cluster intensity with cluster size (here shown as R_g, the radius of gyration) is unaffected.

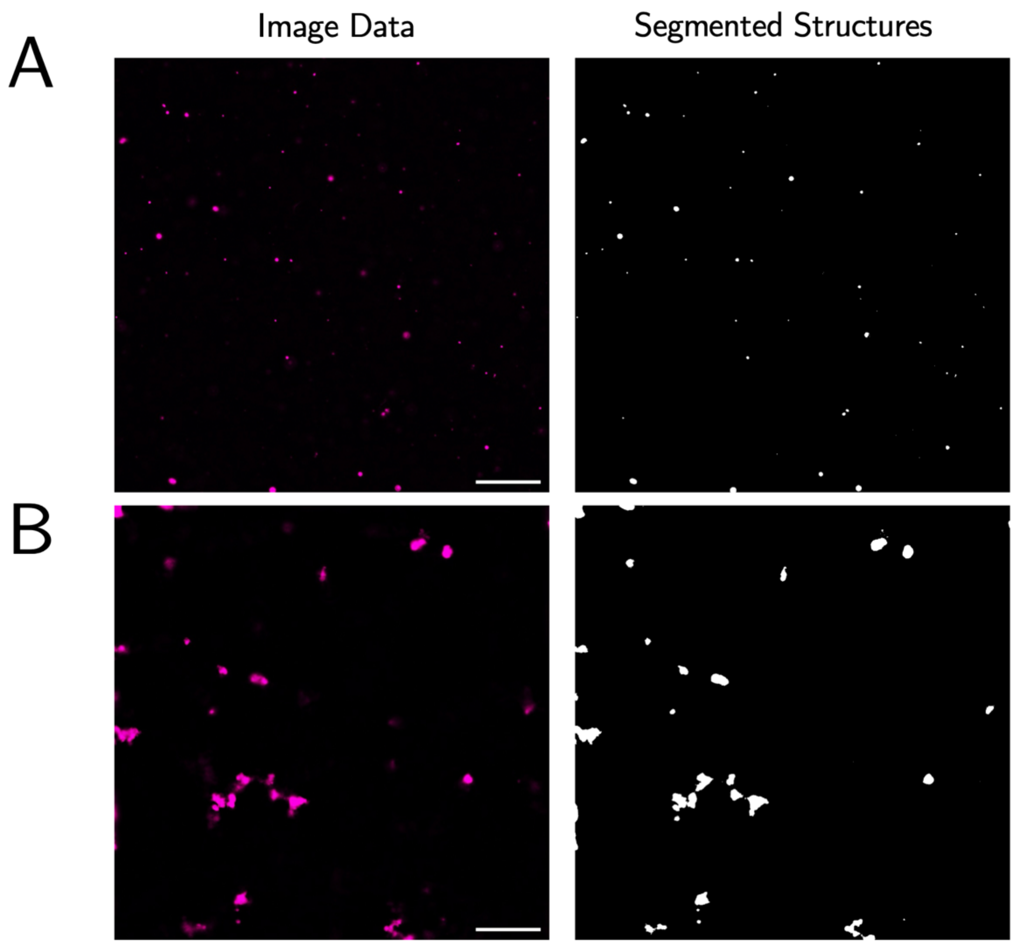

Figure S11. Example segmentation of liposomes and RNA-liposome clusters. (a) Segmentation of liposomes. Left column displays the image data. Right column displays the corresponding structures identified by the algorithm. Scale bar is 20µm. (b) Segmentation of RNA-liposomes 20 minutes after mixing. Left column displays the image data. Right column displays the corresponding structures identified by the algorithm. Scale bar is 20µm.

SI References

1. L. Boltzmann, *Wissenschaftliche Abhandlungen: Bd. 1865-1874* (Chelsea Publishing Company, 1909).
2. J. W. Gibbs, *Elementary Principles in Statistical Mechanics: Developed with Especial Reference to the Rational Foundations of Thermodynamics* (C. Scribner's sons, 1902).
3. E. T. Jaynes, Information Theory and Statistical Mechanics. *Phys. Rev.* **106**, 620–630 (1957).
4. M. Elimelech, J. Gregory, X. Jia, R. A. Williams, "Chapter 6 - Modelling of aggregation processes" in *Particle Deposition & Aggregation*, M. Elimelech, J. Gregory, X. Jia, R. A. Williams, Eds. (Butterworth-Heinemann, 1995), pp. 157–202.
5. T. Matsoukas, The Smoluchowski Ensemble—Statistical Mechanics of Aggregation. *Entropy* **22**, 1181 (2020).
6. R. V. Hogg, E. A. Tanis, D. L. Zimmerman, *Probability and Statistical Inference*, 9th Ed. (Pearson, 2015).
7. M. V. Smoluchowski, Drei Vortrage uber Diffusion, Brownsche Bewegung und Koagulation von Kolloidteilchen. *Zeitschrift fur Physik* **17**, 557–585 (1916).
8. G. Datseris, A. R. Vahdati, T. C. DuBois, Agents.jl: A performant and feature-full agent based modelling software of minimal code complexity. *SIMULATION*, 003754972110688 (2022).
9. R. Jain, K. L. Sebastian, Diffusing diffusivity: Rotational diffusion in two and three dimensions. *The Journal of Chemical Physics* **146**, 214102 (2017).
10. M. A. Zaman, *et al.*, Modeling Brownian Microparticle Trajectories in Lab-on-a-Chip Devices with Time Varying Dielectrophoretic or Optical Forces. *Micromachines* **12**, 1265 (2021).
